# ESF1 and MIPEP proteins promote estrogen receptor-positive breast cancer proliferation and are associated with patient prognosis

**DOI:** 10.1186/s12014-024-09502-8

**Published:** 2024-07-15

**Authors:** Qing Yu, Chunhua Qu, Jinliang Liang, Peiqi Chen, Kaiying Zhang, Yanji Zhang, Yuening Zhang, Zherui Li, Shaojun Liu, Zhaoshou Yang, Hongyan Sun, Anli Yang

**Affiliations:** 1 Department of Clinical Laboratory, Shenzhen Kangning Hospital, Shenzhen, 518000 P. R. China; 2 Department of Clinical Laboratory, Maternal & Child Health Hospital of Foshan, Foshan, 528000 P.R. China; 3 State Key Laboratory of Oncology in South China, Guangdong Provincial Clinical Research Center for Cancer, Sun Yat-sen University Cancer Center, Guangzhou, 510060 P. R. China; 4 Guangdong Key Laboratory of Liver Disease Research, The Third Affiliated Hospital of Sun Yat-Sen University, Guangzhou, 510630 P. R. China; 5 Zhongshan Medical College, Sun Yat-sen University, Guangzhou, 510080 P.R. China; 6 The Third Clinical Medical School, Guangzhou Medical University, Guangzhou, 510180 P.R. China; 7 Guangzhou Institute of Cardiovascular Disease, the Second Affiliated Hospital of Guangzhou Medical University, Guangzhou, 510260 P.R. China; 8 The First Affiliated Hospital, The First Clinical Medicine School of Guangdong Pharmaceutical University, Guangdong Pharmaceutical University, Guangzhou, 510080 P.R. China

**Keywords:** Estrogen receptor-positive breast cancer, ESF1, MIPEP, Proliferation, Prognosis

## Abstract

**Background:**

Estrogen receptor-positive (ER+) breast cancer accounts for two-thirds of all breast cancers, and its early and late recurrences still threaten patients’ long-term survival and quality of life. Finding candidate tumor antigens and potential therapeutic targets is critical to addressing these unmet needs.

**Method:**

The isobaric tags for relative and absolute quantitation (iTRAQ) proteomic analysis was employed to identify the differentially expressed proteins (DEPs) between ER + breast cancer and corresponding adjacent normal tissue. Candidate DEPs were screened by bioinformatic analyses, and their expression was confirmed by immunohistochemical (IHC) staining and western blot. A series of in vitro experiments, including wound healing assay, colony formation, and cell cycle assay, were performed to reveal the functions of selected DEPs. Additionally, their clinical significances were further analyzed.

**Result:**

A total of 369 DEPs (fold change ≥ 2.0 or ≤ 0.66, *P* < 0.05) were discovered. Compared with normal tissue, 358 proteins were up-regulated and 11 proteins were down-regulated in ER + breast cancer. GO and KEGG enrichment analysis showed that DEPs were closely associated with RNA regulation and metabolic pathways. STRING analysis found ESF1 and MIPEP were the hub genes in breast cancer, whose increased expressions were verified by the IHC staining and western blot. Knocking down ESF1 and MIPEP inhibited colony formation and increased cell apoptosis. Besides, knocking down ESF1 inhibited wound healing but not MIPEP. In addition, ESF1 and MIPEP expression were negatively associated with patient prognosis.

**Conclusion:**

The upregulation of ESF1 and MIPEP promoted ER + breast cancer proliferation, which might provide novel targets for the development of new therapies.

**Supplementary Information:**

The online version contains supplementary material available at 10.1186/s12014-024-09502-8.

## Introduction

Breast cancer is the most prevalent malignant tumor in women, contributing to approximately 15.5% of cancer-related fatalities [1]. The diagnosis, treatment, and prognosis of patients with breast cancer are closely related to their molecular subtypes, which are classified by the expression of estrogen receptor (ER), progesterone receptor (PR), human epidermal growth factor receptor 2 (HER2), and proliferation marker Ki67. Estrogen receptor-positive (ER+) breast cancer represents around two-thirds of all breast cancers [2]. It is a highly heterogeneous disease comprising two subtypes with different pathogenesis and distinct prognoses [3].

Postoperative adjuvant endocrine therapy, chemotherapy, and radiotherapy have consolidated the therapeutic effects of patients with ER + breast cancer, but early and late recurrences still occur. There are 20–30% of patients with primary resistance to endocrine therapy, and approximately 40% of patients develop secondary resistance within 10 years of first-line endocrine therapy [4, 5]. Subsequent treatment options are selective estrogen receptor downregulators (SERDs) and CDK4/6 inhibitors. However, the median progression-free survival (mPFS) of patients with advanced ER + breast cancer using the former alone is 16.6 months. Even if the latter is added, mPFS is only extended by about half a year [6, 7]. Moreover, 19.3% of patients who received breast-conserving surgery suffered from local recurrence 10 years after radiotherapy [8]. As for chemotherapy, certain subtypes of breast cancer exhibit a limited response to chemotherapy accompanied by a range of complications [9, 10]. Herein, our future research should focus on the unmet medical needs of patients with ER + tumors.

**Fig. 1:**
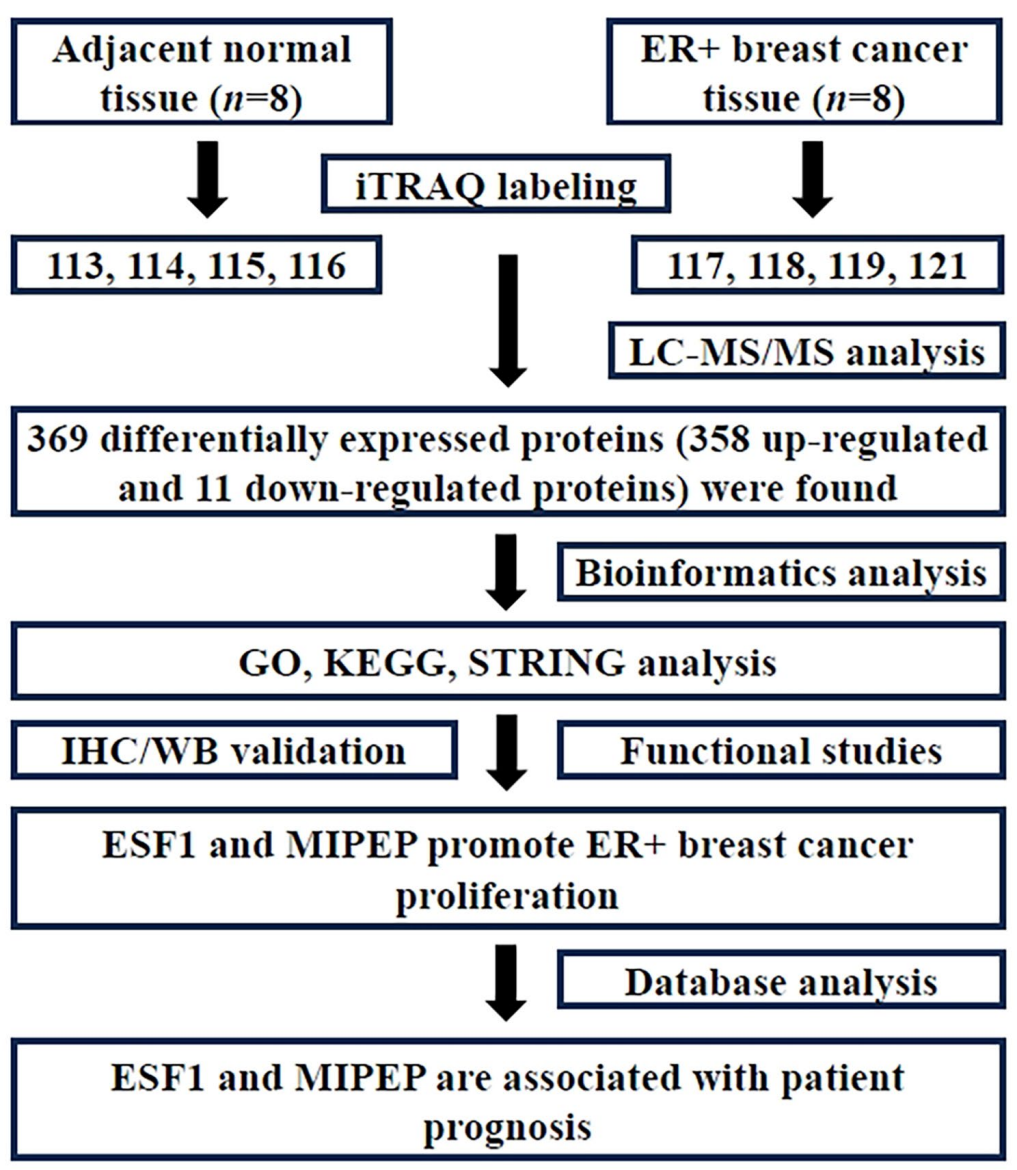
The flow diagram of experimental design. The iTRAQ was applied to identify the differentially expressed proteins in ER + breast cancer. Through bioinformatics analysis and in vitro studies, we found that ESF1 and MIPEP proteins are elevated in ER + breast cancer, promote tumor cell proliferation, and are associated with patient prognosis

The discovery of new tumor antigens and tumorigenesis mechanisms will facilitate the development of new therapeutic targets and methods. However, recent research on breast cancer has predominantly been performed at the genomic and transcriptome levels [11–13]. Proteins, as direct executors of life activities, do not always exhibit expression levels parallel to their transcription levels. In contrast with transcriptome sequencing data, the expression levels of a large number of proteins associated with breast cancer cannot be directly predicted at the messenger RNA (mRNA) level [14]. Protein level analysis could more directly elucidate the biological function of cells. Therefore, the assessment of differential protein expression profiles is crucial for a thorough investigation into the mechanisms underlying the occurrence and progression of breast cancer. In addition, proteomic research on breast cancer in recent years has mainly focused on identifying new molecular subtypes and performing prognosis analysis, while lacking attention to the biological functions of differentially expressed proteins (DEPs), especially in terms of ER + breast cancer [15, 16]. In this study, a quantitative proteomics investigation on ER + breast cancer was conducted using isobaric tags for relative and absolute quantitation (iTRAQ), as depicted in Fig. 1. Through screening DEPs and elucidating their specific functions, we tried to provide a scientific basis for revealing the pathogenesis of ER + breast cancer and seeking potential therapeutic targets.

## Methods

### Collection of clinical samples

Patients were diagnosed with breast cancer by biopsy, and the histopathological type proved to be invasive ductal carcinoma. We confirmed their molecular subtype using the immunohistochemical assay, which exhibited ER positivity (> 1%) and HER-2 negativity (score: 0–1+). Specimens that resulted in central HER2 IHC score of 2 + were subsequently subjected to the fluorescence in situ hybridization (FISH) test for further confirmation [17]. Then patients with a HER-2 score of 2 + but negative FISH result were also included. Tumors with other histopathological types and molecular subtypes were excluded from the study. All samples were obtained from the operation without neoadjuvant therapy. Finally, 50 tissue samples from patients with ER + breast cancer was collected for further proteomics analysis, including 25 cancer tissues and corresponding adjacent normal tissues as control. This study was approved by the Ethics Committee of Sun Yat-sen University Cancer Center (SL-G2023-300-01).

### iTRAQ labeling

The control group (adjacent normal tissue) and ER + breast cancer group each provided eight tissue samples, which were then mixed into four pools respectively. We named control group N1, N2, N3, N4, and ER + breast cancer group T1, T2, T3, T4. Then, protein samples (100 µg) were digested into peptides with trypsin (Promega, USA). Next, iTRAQ labeling was performed using the iTRAQ 8PLEX Multiplex kit (Applied Biosystems Sciex, #4,381,664). Groups N1, N2, N3, N4 and T1, T2, T3, T4 were labeled with iTRAQ reagent individually (including N1-iTRAQ 113 reagent, N2-iTRAQ 114 reagent, N3-iTRAQ 115 reagent, N4-iTRAQ 116 reagent, T1-iTRAQ 117 reagent, T2-iTRAQ 118 reagent, T3-iTRAQ 119 reagent, and T4-iTRAQ 121 reagent) following the instructions of the manufacturer.

### Mass spectrometric analysis

We carried out mass spectrometric analysis as previously reported [18]. The iTRAQ-tagged peptide was reconstituted and loaded onto Phenomenex columns (Gemini-NX 3u C18 110 A; 150*2.00 mm) using a Dionex UltiMate 3000 HPLC system (A-phase: 20mM HCOONH4, PH 10, B-phase: 20mM HCOONH4, 98%CAN, PH 10). After the peptides were eluted with a linear gradient, 15 fractions were collected at 1-minute intervals. Then, we conducted nano LC-MS/MS analysis adopting Q Exactive HF-X (Thermo Scientific). MS data was acquired using a data-dependent top 20 method, the most abundant precursor ions were chosen, and fragmentation with each component was analyzed for 100 min.

### Protein identification and data analysis

PD™ Software 2.4 (Thermo Scientific) was used for protein identification and quantification. Database searching parameters were as follows. Sample type: iTRAQ 8 plex (peptide labeled), Cys alkylation: MMTS, digestion: trypsin, FDR < 1%, First level error: 10ppm, Secondary error: 0.02Da. The paired t-test was adopted to identify statistically significant differences (*P* < 0.05) between breast cancer and controls, with a fold change threshold of ≥ 2.0 or ≤ 0.66. A minimum of two peptide matches in common was considered as differential expression of proteins.

### Bioinformatics analyses

The “clusterProfiler” package of R software was used to conduct the functional enrichment analysis on DEPs, which included KEGG and GO analysis. Enrichment analysis was carried out separately for biological process, molecular function, and cellular component categories. We adjusted the *P* values using the Benjamini - Hochberg (BH) technique. The pathways with an adjusted *P* value and false discovery rate (FDR) below 0.05 were considered statistically significant. Additionally, we set a threshold of Fold Enrichment ≥ 2 and Gene Number ≥ 5. Statistical analysis and ridge mapping were carried out by the “clusterPro” package in R, which is a non-parametric unsupervised analytic method. It is mainly used to determine whether certain metabolic pathways are enriched across samples by analyzing the expression matrix of gene sets. Protein-protein interaction networks of DEPs, including direct and indirect interactions, were analyzed by STRING analysis (http://www.string-db.org). Then we adopted Cytoscape and cytohHubba platform to identify the hub objects, and all parameters were defaulted during this process.

### Immunohistochemical (IHC) staining

After de-paraffinization and rehydration, paraffin slides were heated in sodium citrate buffer to unmask the antigen. 3% hydrogen peroxide was used to block endogen peroxidases. Slides were washed twice, then each section was blocked with 5% BSA for 20 min. After washing the slides 3 times, we draw a circle around the tissue section. Slides were blocked for 15 min each with avidin and biotin. Then, slides were incubated with primary antibody ESF1 (proteintech#23496-1-AP) or MIMEP (proteintech#11002-1-AP) overnight. After incubation with secondary antibodies for 30 min, DAB was incubated for 5 min as substrate. The stained slides were imaged under an optical microscope (NIKON ECLIPSE 80i).

### Cell culture and siRNA transfection

MCF-7 cells (human breast cancer cells) and MCF-10 A cells (normal human breast epithelial cells), identified by STR, were bought from Cell Bank (Shanghai, China). The culture conditions for these cells involved DMEM supplemented with 10% Fetal Bovine Serum (FBS) and 1% Penicillin-Streptomycin Solution (P.S) under 5% CO_2_ and 37 °C. The synthesis of siRNA for ESF1 and MIPEP, as well as the transfection reagents RNAi-Mate, were procured from Genepharma (Shanghai, China). The sequences of siRNAs for ESF1 were 5′-CCCAGAAUCGAGUGUUCUA-3′ and 5′- UAGAACACUCGAUUCUGGG-3′. The sequences of siRNAs for MIPEP were 5′-GGUGCGAGAAGCUGCUUAU-3′ and 5′-AUAAGCAGCUUCUCGCACC-3′. The siRNA powder was dissolved in DEPC-treated water. As instructions provided by the manufacturer, the siRNA was blended with transfection reagents and thoroughly mixed with the resuspended logarithmically growing MCF-7 cells before being seeded onto plates. Cells were collected after a 36-hour transfection period. Their RNAs and proteins were extracted to assess the expression levels of ESF1 and MIPEP in both the control and knockdown groups by qPCR and western blot.

### RT-qPCR

RNA was extracted from the cells using an RNA extraction kit (Accurate Biology, AG21023) 36 h after transfection. Subsequently, the RNA was reverse transcribed into cDNA using the reverse transcription kit (Accurate Biology, AG11728), followed by quantitative PCR using real-time PCR kits (Beyotime, D7260). The primer sequences for the internal control β-actin were 5′-AACACCCCAGCCATGTACGT-3′ and 5′-CCCTCGTAGATGGGCACAGT-3′. For ESF1, the primer sequences were 5′-TGGTAGGACTGCGGACGTAT-3′ and 5′-ATCTCGGGTCCTTTGCAACC-3′. The primer sequences for MIPEP were 5′-GTTGGAGGAAGGGACTGCTC-3′ and 5′-ACTCCAAAAAGACCCCGGC-3′.

### Western blot

The cells were lysed directly by protein lysis buffer (Beyotime, Cat#P0013B, Shanghai, China). Subsequently, protein loading buffer (Beyotime, Cat#P0015, Shanghai, China) was added, and the mixture was boiled for 5 min to prepare the protein loading samples. These protein samples underwent separation using a sodium dodecyl sulfate-polyacrylamide gel electrophoresis (SDS-PAGE) gel and were subsequently transferred onto a polyvinylidene fluoride (PVDF) membrane. The membrane carrying the proteins was blocked using a fast-blocking buffer (Beyotime, Shanghai, China), and then incubated overnight at 4℃ with primary antibodies ESF1 (proteintech#23496-1-AP) or MIPEP (proteintech#11002-1-AP) overnight. Secondary antibodies conjugated with horseradish peroxidase (HRP) were adopted. Following a washing step, the ECL Chemiluminescence Reagent Kit (Beyotime, Shanghai, China) was applied to detect the target protein.

### Wound healing assay

Transfected MCF-7 cells were seeded into 12-well plates until they reached full confluence. Wounds were created using sterile and clean pipette tips, followed by replacing the medium with DMEM containing 1% FBS. Subsequently, digital images were captured using an inverted microscope at 0, 24, 48, and 72 h after medium replacement. The relative migration rate was determined by normalizing the wound area distance measured at 0 h.

### Colony formation assay

Cells were plated at a density of 1500 cells per well in 6-well plates containing 2 mL of DMEM medium supplemented with 10% FBS 48 h after siRNA transfection. After a two-week incubation time at 37 °C, the culture medium was aspirated, and the cells in plates were fixed by ice-cold methanol. Subsequently, cells were stained with crystal violet solution (Beyotime, Cat#C0121, Shanghai, China) for 20 min. We used a camera to capture the photographs of cell-stained images of each well. The ImageJ software was employed to analyze the number and relative area of cell colonies in each well. The number and size of cell colonies in each group were compared with the control group, and their relative colony number and size were calculated.

### Cell cycle and apoptosis assay

Cells were seeded into a 6-well plate 48 h after transfection. They were harvested and resuspended using trypsin without EDTA 24 h after seeding. The cells were divided into two portions, following instructions provided by the manufacturer. One portion was subjected to the cell cycle detection kit (KeyGene, Cat#KGA512, Nanjing, China), while the other portion was processed using the annexin V-AF647/PI apoptosis detection kit (Goonie, Cat#100–102, Guangzhou, China). Flow cytometry (BD FACS Aria II, CA, USA) was adopted to assess the changes in cell cycle arrest and apoptotic cell populations.

### Statistical analysis

Normally or near normally distributed variables were reported as means with standard deviations (SD) and were compared using Student’s t-tests when applicable. Non-normally distributed continuous data were reported as medians with ranges and were compared using Mann–Whitney U-tests. GraphPad Prism 8 (GraphPad Software, USA) was used for all statistical analyses. A *P*-value less than 0.05 was considered statistically significant. ^*^P < 0.05, ^**^P < 0.01, and ^***^P < 0.001.

### Result

#### Clinical characteristics of enrolled patients

All tissue samples were obtained from female patients diagnosed with ER + breast cancer by the Sun Yat-sen University Cancer Center. The patients enrolled in this study ranged from 26 to 80 years old. According to the TNM classification system, all patients were stage I - IIIc with histopathological grade 2–3. By Immunohistochemical staining and fluorescence in situ hybridization (FISH), we confirmed that the collected breast cancer tissue was ER-positive and HER-2-negative (Table 1).

**Table 1:** Clinical characteristics of patients diagnosed with estrogen receptor-positive breast cancer

No.	Gender	Age (yrs)	TNM Stage	HP Grade	ER Positivity	RP Positivity	HER-2	Ki-67	HP Type
1	F	44	I	3	90%	80%	1+	80%	IDC
2	F	39	IIa	3	80%	70%	0	80%	IDC
3	F	26	IIb	3	60%	0	0	80%	IDC
4	F	52	IIa	3	90%	90%	0	70%	IDC
5	F	40	IIa	2	80%	70%	1+	70%	IDC
6	F	45	IIb	3	90%	80%	0	60%	IDC
7	F	34	IIb	2	90%	70%	1+	60%	IDC
8	F	71	IIa	3	60%	0	0	60%	IDC
9	F	46	IIa	2	90%	70%	0	50%	IDC
10	F	33	IIIc	3	90%	40%	1+	50%	IDC
11	F	35	IIa	3	70%	30%	2+ (FISH-)	50%	IDC
12	F	64	IIa	3	95%	95%	2+ (FISH-)	40%	IDC
13	F	47	IIb	2	95%	95%	2+ (FISH-)	40%	IDC
14	F	63	IIb	3	70%	60%	2+ (FISH-)	40%	IDC
15	F	39	IIb	3	95%	50%	1+	35%	IDC
16	F	56	IIb	3	90%	80%	1+	30%	IDC
17	F	80	IIa	2	90%	5%	0	30%	IDC
18	F	58	IIIb	2	95%	95%	2+ (FISH-)	25%	IDC
19	F	48	IIa	2	95%	95%	0	25%	IDC
20	F	44	IIb	3	90%	90%	1+	25%	IDC
21	F	46	IIa	3	90%	90%	1+	25%	IDC
22	F	47	I	2	90%	90%	0	25%	IDC
23	F	36	IIb	2	95%	95%	1+	20%	IDC
24	F	55	IIIc	2	95%	90%	2+ (FISH-)	20%	IDC
25	F	61	IIa	2	90%	60%	2+ (FISH-)	20%	IDC
26	F	36	IIIc	2	95%	50%	2+ (FISH-)	20%	IDC
27	F	45	I	2	95%	40%	1+	20%	IDC
28	F	63	IIa	2	95%	10%	2+ (FISH-)	20%	IDC
29	F	44	IIa	2	95%	90%	2+ (FISH-)	15%	IDC
30	F	54	IIa	2	90%	80%	1+	15%	IDC
31	F	37	IIa	2	90%	80%	1+	15%	IDC
32	F	43	IIa	3	90%	90%	2+ (FISH-)	40%	IDC
33	F	38	IIa	2	75%	70%	1+	20%	IDC

F, female; HP, Histopathological; FISH, fluorescence in situ hybridization; IDC, invasive ductal carcinoma

### Differential expression proteins screened by iTRAQ proteomics

**Fig. 2:**
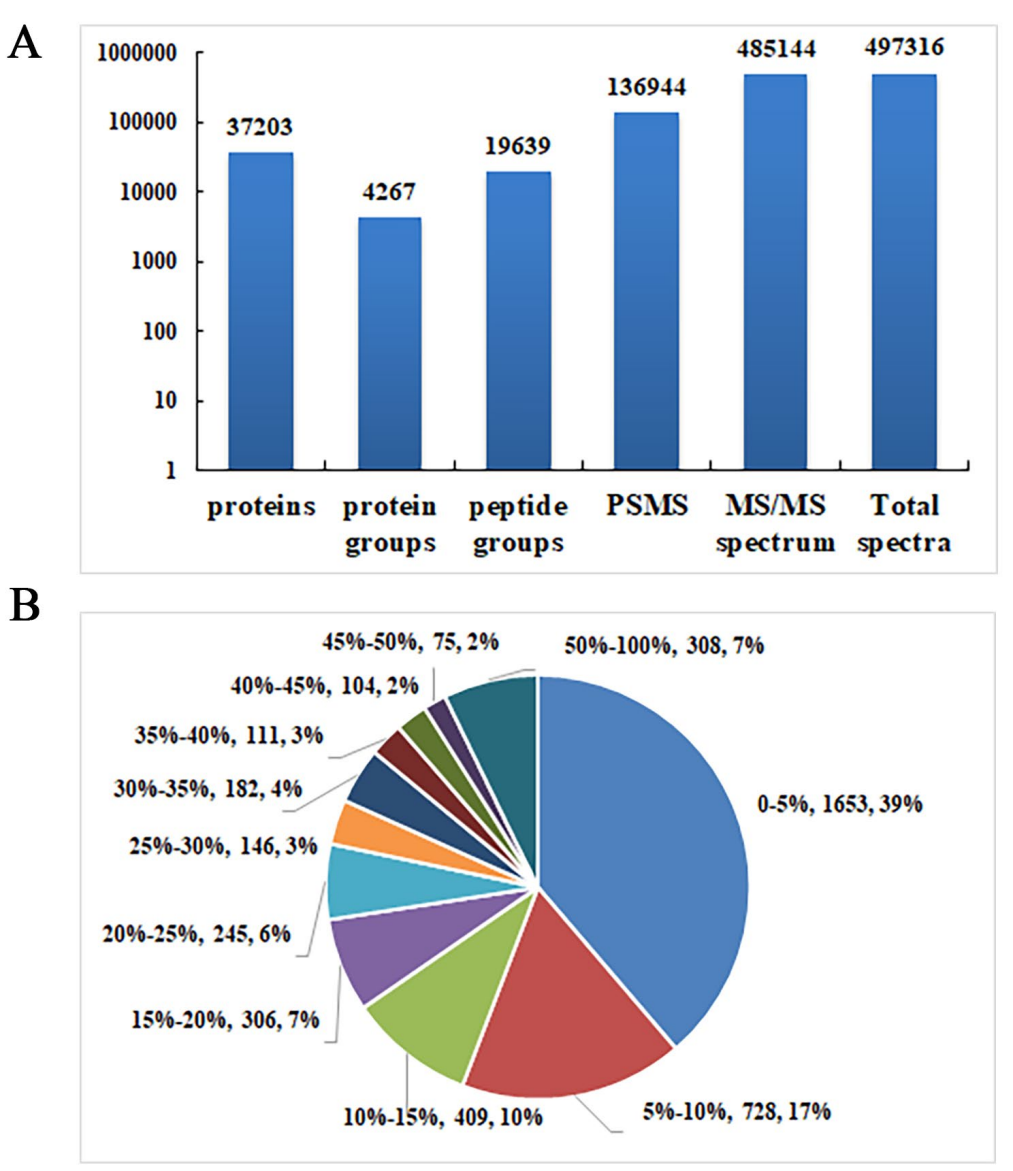
Proteomic profiling of ER + breast cancer. **(A)** Number of identified peptides and proteins acquired from iTRAQ analysis. **(B)** Identified proteins were classified based on the percent coverage of protein sequences, displayed as follows. Percent coverage of protein sequences (%), number of proteins (*n*), total proportion (%)

The iTRAQ labeling identified a total of 136,944 peptide spectrum matches (PSMs), which were present in 37,203 proteins. Due to the presence of common peptide sequences among certain proteins, it was necessary to group them based on sequence homology and isoforms, resulting in the identification of 4267 protein groups (Fig. 2A). Continuing the analysis of protein sequence coverage, 308 proteins exhibited coverage between 50 and 100%, 472 proteins between 30 and 50%, 1106 proteins between 10 and 30%, and 2381 proteins below 10% (Fig. 2B). According to the criteria of ratio fold change ≥ 2.0 or ≤ 0.66, it was defined as the differential expression protein of interest when its *P* < 0.05. Compared with adjacent normal tissue, there were 358 proteins up-regulated and 11 proteins down-regulated in ER + breast cancer tissue (Supplementary Table 1).

### The GO and KEGG analysis of differential expression proteins

**Fig. 3:**
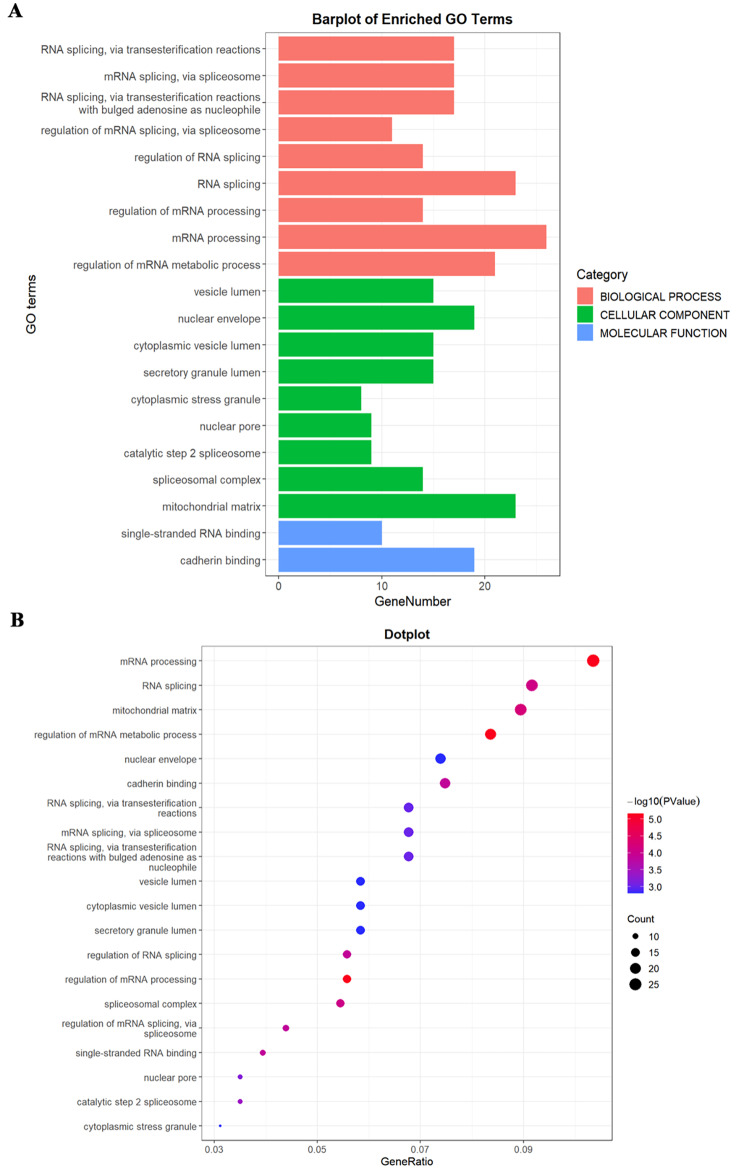
The GO and KEGG analysis of differentially expressed proteins in ER + breast cancer. **(A)** Gene Ontology analysis revealed the enrichment of differentially expressed proteins in biological processes, cellular components, and molecular function categories. **(B)** The KEGG analysis showed signaling pathway enrichment. Circle area represented the number of differentially expressed proteins in each pathway term, and color represented the adjusted *P*-value

We conducted GO (Gene Ontology) and KEGG (Kyoto Encyclopedia of Genes and Genomes) analysis on differentially expressed proteins. GO analysis included three dimensions: biological processes (BP), cellular components (CC), and molecular function (MF). In the BP group, differentially expressed proteins were mainly enriched in mRNA processing, RNA splicing, and their regulation. Certain proteins particularly accumulated in mitochondrial matrix and nuclear envelope within the CC category. At the same time, related proteins mainly aggregated in cadherin binding and single-stranded RNA binding in the MF group (Fig. 3A). The KEGG analysis result indicated that differentially expressed proteins were mainly enriched in mRNA processing and metabolic regulation pathways (Fig. 3B). Notably, significantly upregulated proteins related to metabolism included phosphomevalonate kinase, ribokinase, phosphopentomutase, and glyceraldehyde-3-phosphate dehydrogenase.

### STRING analysis selected candidate proteins

**Fig. 4:**
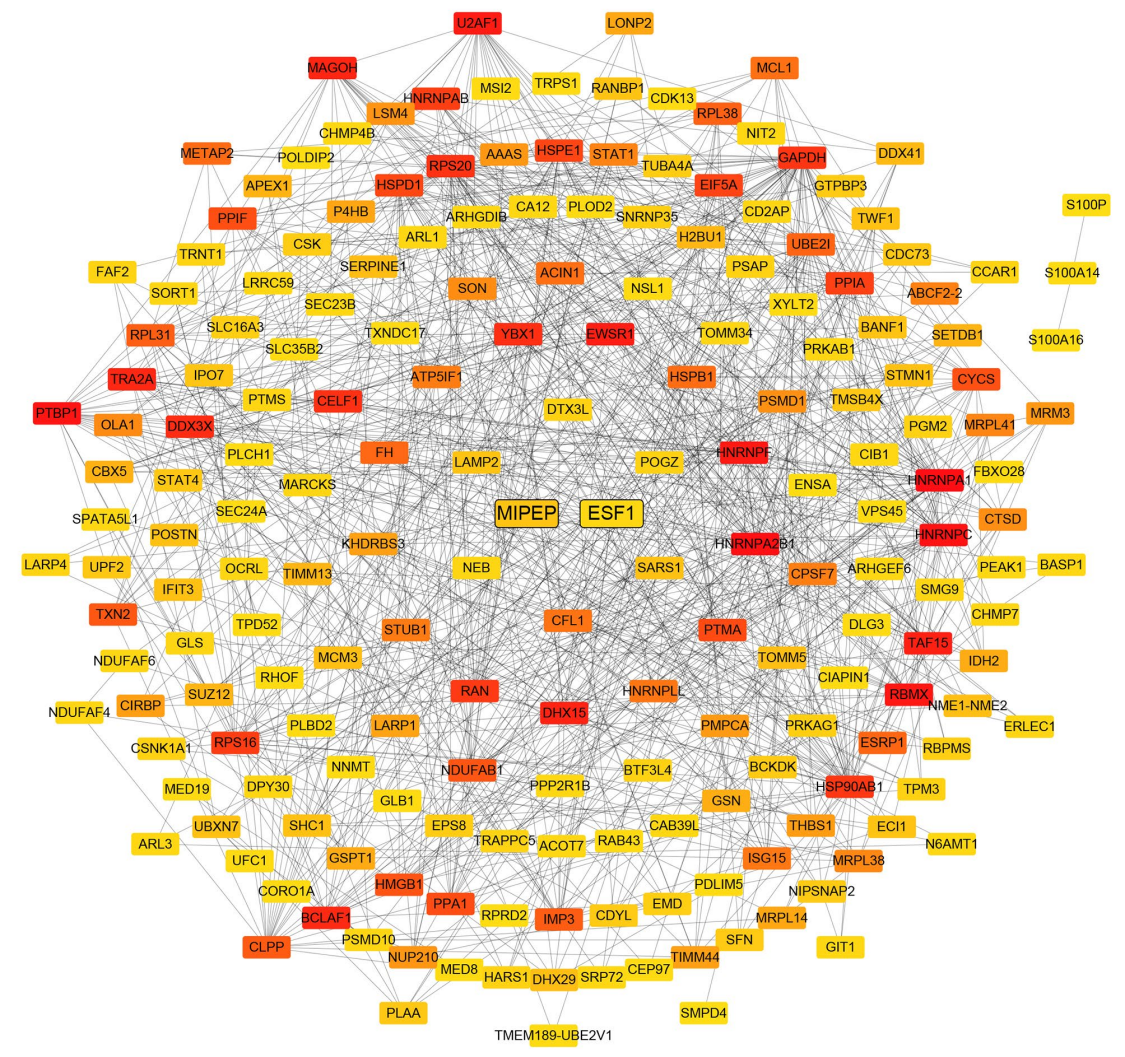
The protein-protein interaction network analysis. The top 200 hub proteins were displayed. Nodes are proteins, and lines represent functional associations between proteins. Red indicates that the protein is more functionally related to other proteins

Biological processes are regulated through complex network systems in which components interact with each other through various pathways. STRING analysis of differentially expressed proteins was constructed to better understand ER + breast cancer’s pathogenesis. The top 200 hub proteins proteins were displayed (Fig. 4). As could be seen from the network diagram, numerous proteins were distributed at the intersections of core transportation hubs, such as ESF1 (ESF1 nucleolar pre-rRNA processing protein homolog), MIPEP (mitochondrial intermediate peptidase), TMEM24 (C2 calcium dependent domain containing 2 like, also named C2CD2L), and SART1 (Spliceosome associated factor 1 recruiter of U4/U6.U5 tri-snRNP). This indicated that they might play an important role in the development of ER + breast cancer. To identify novel tumor antigens and potential therapeutic targets, the functions of candidate proteins were further analyzed through PubMed literature retrieval. We found that the ESF1 and MIPEP proteins have never been reported in the field of breast cancer research.

### ESF1 and MIPEP expression upregulated in ER + breast cancer

**Fig. 5:**
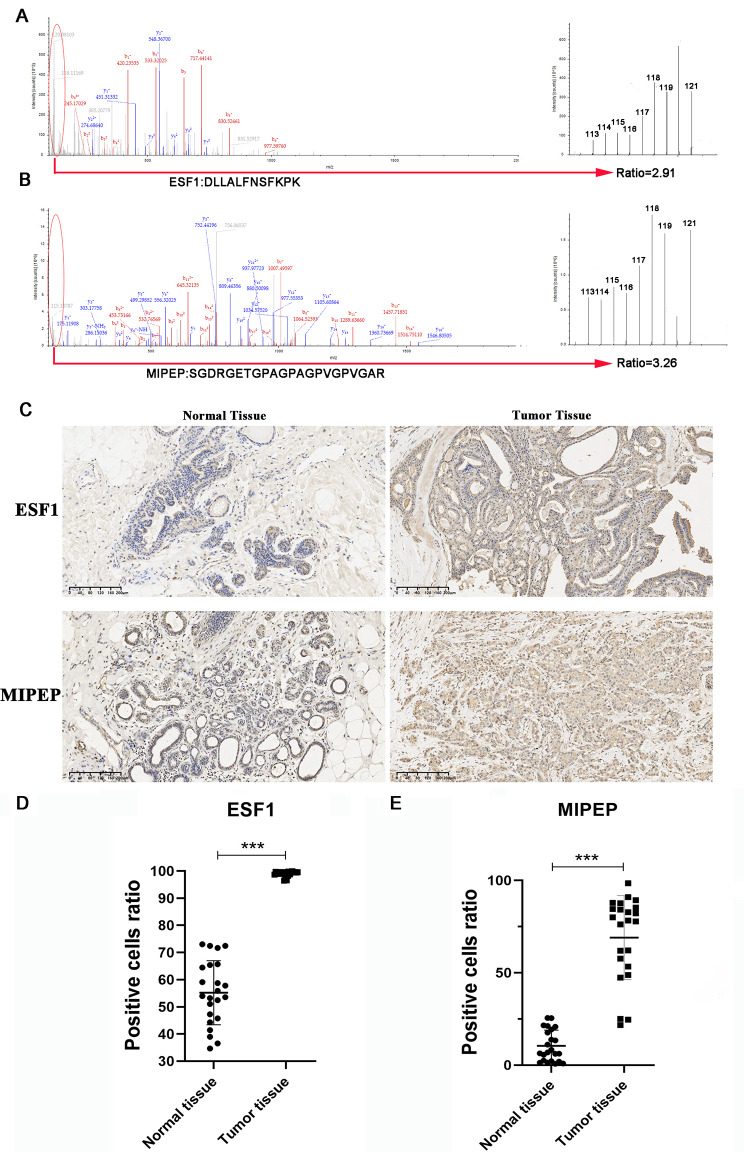
Verification of ESF1 and MIPEP expression in human breast cancer tissue. **(A-B)** A representative MS/MS spectrum indicated important peptide segments for ESF1 and MIPEP. iTRAQ tags showed the relative expression of these proteins individually in ER + breast cancer compared to the control. **(C)** Representative images of ESF1 and MIPEP immunohistochemical staining in ER + breast cancer tissue. Scale bars, 200 μm. (representative images are from 25 pairs of tissue sections). **(D-E)** Statistical analysis of ESF1 and MIPEP expressions in breast cancer tissues compared to corresponding adjacent normal tissue. Statistical analysis was performed using the paired t-test. All data were represented as mean ± s.d. (*n* = 25 per group). ^***^P < 0.001

To further confirm the expression of candidate proteins, iTRAQ quantification in the MS/MS spectrogram was analyzed. We discovered that both ESF1 and MIPEP were upregulated 2.91-fold and 3.26-fold in the ER + breast cancer group, respectively (*P* < 0.01) (Fig. 5A-B). Subsequently, we conducted IHC staining to confirm the expression of ESF1 and MIPEP (Fig. 5C). 25 pairs of tissue sections derived from patients with ER + breast cancer were included. Compared with the adjacent normal tissue group, the expression of ESF1 in the ER + breast cancer group was increased by 1.91 times (*P* < 0.001) (Fig. 5D). Similarly, MIPEP demonstrated a 2.92-fold upregulation in ER + breast cancer tissue (*P* < 0.001) (Fig. 5E). The expression of ESF1 and MIPEP proteins was further validated in an independent cohort (CPTAC dataset, https://ualcan.path.uab.edu/analysis-prot.html) (Supplementary Fig. 1A-B). These results indicated that the expression of ESF1 and MIPEP was upregulated in ER + breast cancer.

### ESF1 and MIPEP promote breast cancer cell proliferation

**Fig. 6:**
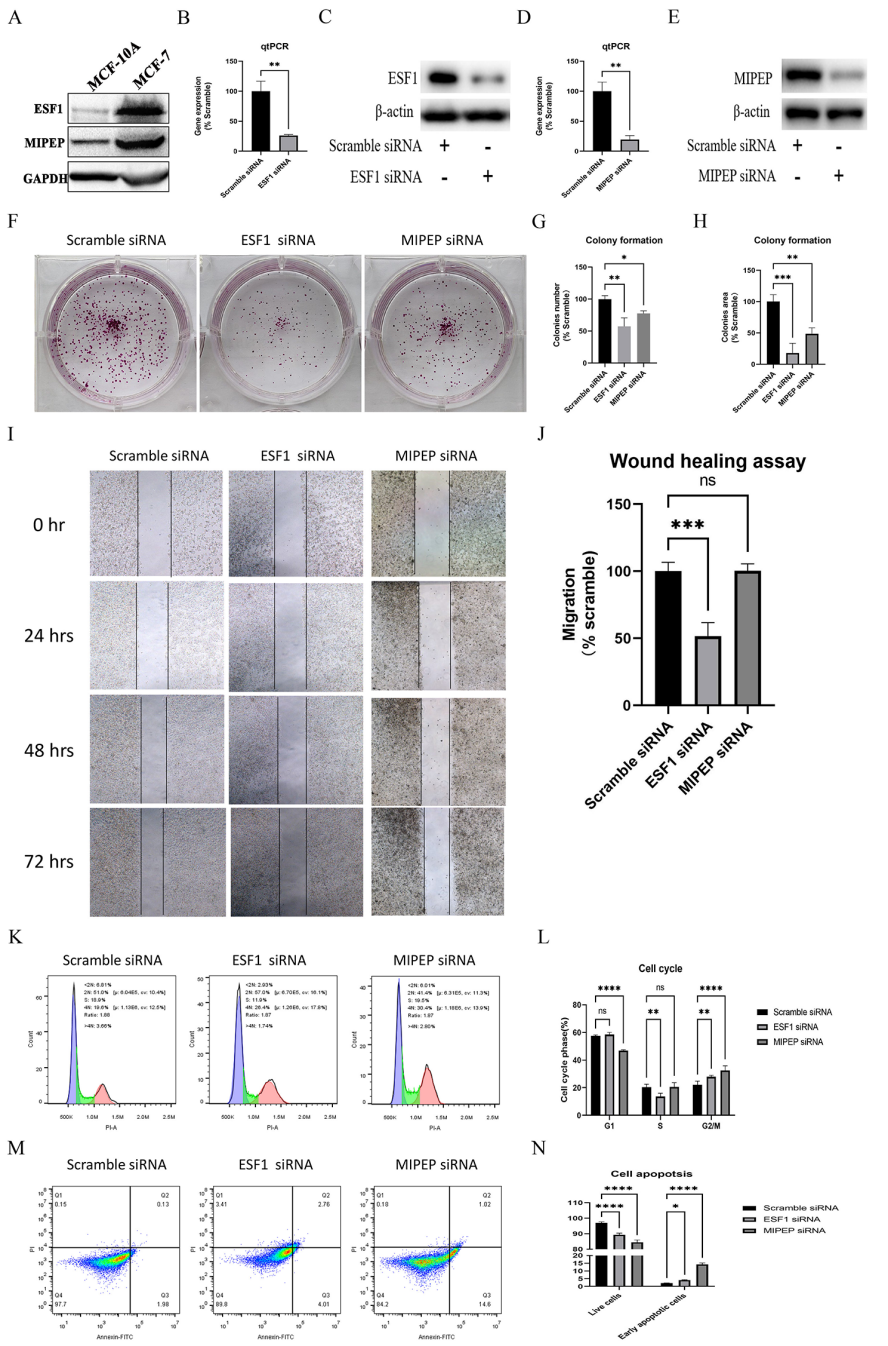
Functional effects of ESF1 and MIPEP on breast cancer cells. **(A)** The ESF1 and MIPEP proteins expression in human breast cancer cell line MCF-7 and human normal breast epithelial cell line MCF-10 A assessed by western blot. **(B-C)** The expression of ESF1 was verified by real-time PCR (B) and western blot (C) after ESF1 siRNA transfection in MCF-7 cells (*n* = 3 independent experiments). (D-E) The expression of MIPEP was tested by real-time PCR **(D)** and western blot **(E)** after MIPEP siRNA transfection in MCF-7 cells (*n* = 3 independent experiments). **(F-H)** The colony formation assay of MCF-7 cells **(F)**. The relative number **(G)** and relative area **(H)** of colonies in different groups were calculated and compared with the scramble group (*n* = 3 independent experiments). **(I-J)** The wound healing assays of MCF-7 cells (*n* = 3 independent experiments). Digital images were captured at 0, 24, 48, and 72 h by using an inverted microscope (I). Compared with the scramble group, cell migration rates of different groups were quantified **(J)**. **(K-L)** The cell cycle assay of MCF-7 cells (*n* = 3 independent experiments). The represented results in each group **(K)**. Comparison of cell percentage in the G1 and S phases among different groups **(L)**. **(M-N)** The cell apoptosis assay of MCF-7 cells (*n* = 3 independent experiments). The represented results of detection in each group **(M)**. Comparison of normal cells (Q1 region) and early apoptotic cells (Q3 region) among different groups **(N)**. Statistical analysis was performed using the paired t-test. All data were represented as mean ± s.d. ns, not significant. ^*^P < 0.05. ^**^P < 0.01. ^***^P < 0.001. ^****^P < 0.0001

To better understand the functions of ESF1 and MIPEP in ER + breast cancer, we performed a series of in vitro experiments. Firstly, we confirmed that ESF1 and MIPEP expression were upregulated in MCF-7 cells compared with MCF-10 A cells (Fig. 6A). After siRNA transfection, ESF1 gene and protein expression decreased dramatically in MCF-7 cells, which were detected by RT-qPCR and western blot (Fig. 6B-C). Similarly, a significant reduction in MIPEP gene and protein expression was observed post-transfection (Fig. 6D-E). The number and size of cell colonies decreased obviously after ESF1 and MIPEP knockdown, which were verified by the colony formation assay (Fig. 6F-H). In the wound healing assay, ESF1 knockdown compromised the wound healing rate, whereas MIPEP knockdown showed no significant changes (Fig. 6I-J). Besides, cell cycle analysis revealed that the S phase cells in the ESF1 knockdown group reduced significantly, while the G1 phase cells in the MIPEP knockdown group decreased obviously (*P* < 0.01) (Fig. 6K-L). In addition, the knockdown of ESF1 and MIPEP resulted in increased cell apoptosis rates (*P* < 0.05) (Fig. 6M-N). These findings proved that ESF1 and MIPEP played a crucial role in promoting breast cancer cell proliferation.

### ESF1 and MIPEP are associated with patient prognosis

**Fig. 7:**
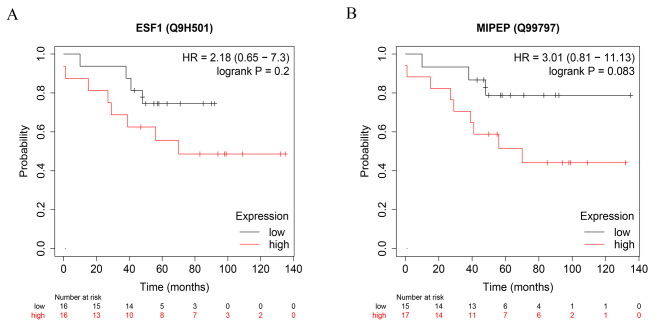
The clinical value of ESF1 and MIPEP proteins. **(A)** Kaplan-Meier plot of overall survival (OS) between patients with high and low ESF1 protein expression. **(B)** Kaplan-Meier plot of OS between patients with high and low MIPEP protein expression. Statistical analysis was performed using the log-rank test (*n* = 32)

To clarify the clinical significance of ESF1 and MIPEP, we searched and analyzed the online database, such as the Kaplan Meier (KM) plotter. We found that patients with higher ESF1 protein expression were inclined to have worse overall survival (OS) (Fig. 7A). The high MIPEP protein expression group tended to have a shorter OS period compared with the low expression group (*P* = 0.08) (Fig. 7B). Due to the limited sample size of the online database, no significant difference was found between the high and low expression groups. However, the curves of the two groups were separated clearly, indicating that the expression of ESF1 and MIPEP proteins might be related to patient’s prognosis.

## Discussion

Despite the development of comprehensive treatments, ER + breast cancer still threatens the lives of two-thirds of breast cancer patients due to early and late recurrence. Proteomic analysis helps identify potential effective clinical markers and drug targets. To further improve their prognosis and quality of life, we adopted iTRAQ-based quantitative proteomics technology to screen the differentially expressed proteins (DEPs) between ER + breast cancer and corresponding adjacent normal tissue. In our study, 369 DEPs (358 upregulated and 11 downregulated) were discovered.

Among them, 252 DEPs have been reported in previous studies. For example, ubiquitin-associated protein 2-like (UBAP2L) is the most significantly upregulated protein in ER + breast cancer tissue (fold change 5.696, *P* < 0.01). He et al. reported that UBAP2L expression was significantly upregulated in both breast cancer tissue and cell lines. Gene knockout experiments demonstrated that UBAP2L inhibition could impede cell proliferation and lead to cell cycle arrest at the G2/M phase, suggesting UBAP2L is a potential target for breast cancer treatment [19].

Most of the DEPs identified via GO analysis were associated with RNA regulation and metabolic pathways. Take for an example, the cell division cycle and apoptosis regulator protein 1 (CARP-1) was significantly upregulated in ER + breast cancer tissue (fold change 2.834, *P* < 0.01), indicating the active proliferation and division capabilities of tumor cells. As a perinuclear phosphoprotein, CARP-1 is a coactivator of the steroid/thyroid nuclear receptors β-catenin and P53, thereby dynamically regulating cell growth and apoptosis [20]. CARP-1 is associated with estrogen-dependent growth of breast cancer cells and regulates the expression of key proliferation-related genes, which correlates with chemosensitivity to doxorubicin (ADR) [21].

KEGG analysis showed that DEPs were mainly enriched in metabolic pathways, suggesting that metabolic remodeling was closely related to the occurrence of breast cancer [22, 23]. There were 20 upregulated proteins associated with metabolic pathways, including phosphomevalonate kinase (PMVK), ribokinase, and phosphoglucomutase-2 (PGM2), which was consistent with Asleh K’s report [15]. In our study, their expression upregulated significantly (fold change > 2, *P* < 0.01). Taking PMVK as an example, it is a target of miR-874, which hindered the mevalonate pathway by depleting geranylgeranyl pyrophosphate (GGPP), subsequently activating the P53 pathway and promoting breast cancer cell apoptosis [24].

During STRING analysis, we found ESF1, MIPEP, TMEM24, and SART1 were the hub genes in ER + breast cancer’s pathogenesis. TMEM24 is a lipid-binding protein that delivers ER-synthesized phosphatidylinositol to the plasma membrane, thereby promoting the activity of downstream cell proliferation-related PI3K-AKT-mTOR signaling pathway [25, 26]. SART1 is a spliceosome protein that mediates the degradation of the HIF1-α protein and facilitates the proliferation of renal cell carcinoma (RCC) cells [27, 28]. Under the stimulations of extracellular signals such as cytokines, STAT1 can be phosphorylated by receptor-associated kinases and translocated to the nucleus to act as a transcription activator [29]. Inflammatory mediators such as interferon-γ can cause an increase in oxidative stress in a STAT1-dependent manner, thereby enhancing the antitumor effect of phenformin [30]. Hence, the elevated expression of TMEM24 and SART1 may be linked to increased cell proliferation in breast cancer.

As the novel proteins discovered in ER + breast cancer, the functions of ESF1 and MIPEP were further discussed and verified in this study. ESF1 is a nuclear protein conserved from yeast to mammals, involving in the biosynthesis of ribosomal RNA (rRNA) [31]. rRNA binds to proteins to form ribosomes and synthesizes amino acids into peptide chains under the guidance of mRNAs [32]. ESF1 mutations can lead to cell death by upregulating the P53 signaling pathway and cause pharyngeal cartilage defects in a zebrafish model [33]. These findings indicated a crucial role for the ESF1 protein in cell survival and proliferation, potentially promoting tumor cell proliferation by inhibiting the P53 pathway. ESF1 protein plays a prominent role in predicting the recurrence of hepatitis B virus-related hepatocellular carcinoma and the response to gastric cancer chemotherapy [34, 35]. However, whether ESF1 overexpression promotes tumorigenesis has not been reported. In this study, an upregulated expression of ESF1 was observed for the first time in both breast cancer tissue and the MCF-7 breast cancer cell line. ESF1 promoted the colony formation, accelerated the cell cycle, and enhanced the migration ability of breast cancer cells. These findings strongly indicated that ESF1 had the potential to enhance the proliferation and metastasis of breast cancer cells.

MIPEP is located in the mitochondrial matrix, where it cleaves proteins entering the mitochondria to regulate the maturation of proteins related to the oxidative phosphorylation (OXPHOS) system [36]. The mutations in MIPEP affect mitochondrial protein homeostasis, causing cardiomyopathy and aging, even contributing to lung cancer susceptibility [37–39]. MIPEP participates in the proteolysis of the Notch receptor and bioactivation of anticancer compounds imipridone [40, 41]. Karol et al. analyzed the gene expression profiles of canine breast cancer patients with different malignancies and reported a gradual increase in the expression level of MIPEP with escalating malignancy [42]. The upregulation of MIPEP expression may promote cell proliferation by enhancing the energy metabolism of breast cancer cells. Our study revealed for the first time that MIPEP was upregulated in human breast cancer tissue and could promote the proliferation of MCF-7 cells by promoting colony formation and accelerating the cell cycle, without exerting an impact on cell migration ability. MIPEP appeared to specifically influence the process of proliferation, which may serve as a potential therapeutic target for breast cancer treatment.

However, there are still some limitations of our current study. Firstly, although we discovered two novel proteins in ER + breast cancer development, their underlying molecular mechanisms remain unclear and require further exploration. Besides, the expression and clinical significance of ESF1 and MIPEP should be validated in other cohorts with a large sample size. Although their expressions were likely associated with poor patient outcomes, this result had not been verified in a well-designed multicenter study.

In conclusion, our study employed the iTRAQ-based quantitative proteomics method to identify many differentially expressed proteins between ER + breast cancer and the adjacent normal tissue. Subsequent immunohistochemical staining validated the significant upregulation of ESF1 and MIPEP in the ER + breast cancer tissue sections. Furthermore, a series of experiments conducted on the MCF-7 cell line revealed that both ESF1 and MIPEP could promote the proliferation of breast cancer cells. In addition, we found that ESF1 and MIPEP were negatively associated with patient prognosis. Therefore, ESF1 and MIPEP proteins played a considerable role in ER + breast cancer development, which might serve as potential therapeutic targets.

### Electronic supplementary material

Below is the link to the electronic supplementary material.

Supplementary Material 1

Supplementary Material 2: Fig. 1. Validation of ESF1 and MIPEP proteins expression in an independent cohort. (A) The expression of ESF1 in normal breast tissue (*n* = 18) and three major molecular subtypes, including luminal ones (*n* = 64). (B) The expression of MIPEP in normal breast tissue (*n* = 18) and major subtypes, including luminal ones (*n* = 64). Z-values represent standard deviations from the median across samples for the given cancer. Log2 Spectral count ratio values from CPTAC were first normalized within each sample profile, then normalized across samples.

## Data Availability

No datasets were generated or analysed during the current study.
