# Supplementary material for: ESF1 and MIPEP proteins promote estrogen receptor-positive breast cancer proliferation and are associated with patient prognosis
Source: Clin Proteomics. 2024 Jul 15;21:50. doi: 10.1186/s12014-024-09502-8 (PMC11247778; doi:10.1186/s12014-024-09502-8)
Supplement: Supplementary file 1 — Supplementary Material 1 [file 12014_2024_9502_MOESM1_ESM.docx]

**Supplementary Table 1**. Expression of differential expression proteins between estrogen receptor-positive breast cancer and corresponding adjacent normal tissue.

| N | Accession | Protein Name | Protein Coverage (%) | Ratio S/C | *P*-value |
| --- | --- | --- | --- | --- | --- |
| 1 | B4DZJ6 | Ubiquitin-associated protein 2-like | 2 | 5.696 | 0.009547 |
| 2 | A0A2R8Y7H5 | Regulator of telomere elongation helicase 1 | 40 | 4.250 | 0.003333 |
| 3 | O14874 | 3-methyl-2-oxobutanoate dehydrogenase kinase, mitochondrial | 4 | 3.922 | 0.024955 |
| 4 | Q96K17 | Transcription factor BTF3 homolog 4 | 19 | 3.751 | 0.010197 |
| 5 | Q92804 | TATA-binding protein-associated factor 2N | 5 | 3.699 | 0.003896 |
| 6 | E5RG12 | KH domain-containing, RNA-binding, signal transduction-associated protein 3 | 4 | 3.442 | 0.029570 |
| 7 | O14523 | Phospholipid transfer protein C2CD2L | 1 | 3.336 | 0.000318 |
| 8 | B2RCT7 | solute carrier family 2 | 1 | 3.324 | 0.001487 |
| 9 | B3KQ71 | Golgi phosphoprotein 3 | 19 | 3.297 | 0.005544 |
| 10 | Q99797 | Mitochondrial intermediate peptidase | 3 | 3.260 | 0.009352 |
| 11 | Q9UJV9 | Probable ATP-dependent RNA helicase DDX41 | 2 | 3.209 | 0.005044 |
| 12 | B4DRF6 | DNA helicase | 3 | 3.187 | 0.014269 |
| 13 | Q59GA5 | Insulysin variant | 3 | 3.177 | 0.016972 |
| 14 | J3KT73 | 60S ribosomal protein L38 | 20 | 3.114 | 0.015325 |
| 15 | J3KRU7 | Transmembrane protein 94 | 11 | 3.075 | 0.000895 |
| 16 | A0A1S5UZH2 | F-actin monooxygenase | 1 | 3.024 | 0.000951 |
| 17 | B4DDH9 | U4/U6.U5 tri-snRNP-associated protein 1 | 2 | 3.020 | 0.000684 |
| 18 | B4DNL8 | Galactosylgalactosylxylosylprotein 3-beta-glucuronosyltransferase | 4 | 2.962 | 0.023709 |
| 19 | Q9C005 | dpy-30 | 45 | 2.927 | 0.007852 |
| 20 | H7C1M2 | Protein SON | 3 | 2.922 | 0.002321 |
| 21 | A0JLU5 | ESF1 protein | 2 | 2.910 | 0.002125 |
| 22 | Q9HC36 | rRNA methyltransferase 3, mitochondrial | 6 | 2.904 | 0.014823 |
| 23 | A0A3G5BD32 | MHC class I antigen | 16 | 2.887 | 0.018230 |
| 24 | B7Z5L6 | Poly (ADP-ribose) polymerase 9 | 4 | 2.884 | 0.017632 |
| 25 | Q969Y2 | tRNA modification GTPase GTPBP3, mitochondrial | 1 | 2.858 | 0.024783 |
| 26 | Q6FGV9 | Phosphomevalonate kinase | 4 | 2.845 | 0.006127 |
| 27 | Q8IX12 | Cell division cycle and apoptosis regulator protein 1 | 3 | 2.834 | 0.001622 |
| 28 | A0A024QYT5 | Serpin peptidase inhibitor | 8 | 2.834 | 0.009073 |
| 29 | Q14146 | Unhealthy ribosome biogenesis protein 2 | 2 | 2.809 | 0.016805 |
| 30 | B8ZZ54 | 10 kDa heat shock protein, mitochondrial | 85 | 2.806 | 0.002027 |
| 31 | E9PL50 | Syntabulin | 6 | 2.798 | 0.003074 |
| 32 | A0JLT2 | Mediator of RNA polymerase II transcription subunit 19 | 10 | 2.788 | 0.005654 |
| 33 | F6TB26 | Tudor and KH domain-containing protein (TDRKH) | 5 | 2.766 | 0.014022 |
| 34 | B3KP18 | RAD50-interacting protein 1 (Rint1) | 3 | 2.735 | 0.008040 |
| 35 | Q53S24 | Prothymosin | 13 | 2.731 | 0.003056 |
| 36 | F6IR49 | MHC class I antigen | 7 | 2.713 | 0.005746 |
| 37 | B2RB07 | Ubiquitin carboxyl-terminal hydrolase | 4 | 2.711 | 0.003165 |
| 38 | Q6P1J9 | Parafibromin | 6 | 2.695 | 0.009013 |
| 39 | A0A024RBU8 | Patatin-like phospholipase domain containing 4 (PNPLA4) | 6 | 2.695 | 0.028102 |
| 40 | Q96FQ6 | S100-A16 | 41 | 2.673 | 0.002477 |
| 41 | Q8WUK0 | Phosphatidylglycerophosphatase and protein-tyrosine phosphatase 1 | 6 | 2.671 | 0.029699 |
| 42 | Q8IW35 | Centrosomal protein of 97 kDa | 3 | 2.660 | 0.009550 |
| 43 | A0A2R8Y7W5 | Cyclin-dependent kinase 13 | 2 | 2.646 | 0.006147 |
| 44 | Q10713 | Mitochondrial-processing peptidase subunit alpha | 14 | 2.625 | 0.003196 |
| 45 | Q9Y2S7 | Polymerase delta-interacting protein 2 | 3 | 2.621 | 0.004222 |
| 46 | Q8TD06 | Anterior gradient protein 3 | 44 | 2.616 | 0.010843 |
| 47 | P62328 | Thymosin beta-4 | 45 | 2.576 | 0.000653 |
| 48 | P06127 | T-cell surface glycoprotein CD5 | 2 | 2.575 | 0.002122 |
| 49 | Q6ZQZ4 | Actin cross-linking family protein 7 | 1 | 2.575 | 0.001674 |
| 50 | A0A384N6A4 | 10 kDa heat shock protein, mitochondrial | 77 | 2.572 | 0.001162 |
| 51 | Q6FH49 | NNMT protein | 7 | 2.566 | 0.001741 |
| 52 | Q8NE02 | AKAP8 protein | 3 | 2.564 | 0.009162 |
| 53 | Q9Y5N5 | Methyltransferase N6AMT1 | 4 | 2.542 | 0.001251 |
| 54 | Q9P2P1 | Protein NYNRIN | 1 | 2.541 | 0.016180 |
| 55 | Q8IXM3 | 39S ribosomal protein L41, mitochondrial | 7 | 2.538 | 0.004197 |
| 56 | B7ZAC7 | Nucleosome assembly protein 1-like 4 | 10 | 2.534 | 0.014740 |
| 57 | Q9HAU5 | Regulator of nonsense transcripts 2 | 2 | 2.531 | 0.019984 |
| 58 | H0YIC9 | 5'-AMP-activated protein kinase subunit gamma-1 | 8 | 2.530 | 0.002903 |
| 59 | K7EKP8 | Cytosolic acyl coenzyme A thioester hydrolase | 4 | 2.526 | 0.009156 |
| 60 | Q5TEJ8 | Protein THEMIS2 | 3 | 2.523 | 0.002227 |
| 61 | A0A2X0SFB0 | ARHGEF11 | 0 | 2.516 | 0.044089 |
| 62 | B4DRJ1 | 2-hydroxyphytanoyl-CoA lyase | 3 | 2.498 | 0.017169 |
| 63 | E7EN44 | Lon protease homolog 2, peroxisomal | 4 | 2.496 | 0.007781 |
| 64 | Q8N6C5 | Immunoglobulin superfamily member 1 | 1 | 2.478 | 0.045745 |
| 65 | B2R8W8 | Rab-3 | 19 | 2.462 | 0.000678 |
| 66 | P29353 | SHC-transforming protein 1 | 4 | 2.443 | 0.019419 |
| 67 | A0A024RBU2 | U1 snRNP-binding protein homolog | 6 | 2.437 | 0.004584 |
| 68 | Q8TDB6 | E3 ubiquitin-protein ligase DTX3L | 1 | 2.426 | 0.007365 |
| 69 | Q96NB2 | Sideroflexin-2 | 13 | 2.424 | 0.001906 |
| 70 | B7Z592 | Zinc finger CCCH domain-containing protein 15 | 2 | 2.419 | 0.014567 |
| 71 | M0R1P3 | Kelch-like protein 26 | 11 | 2.418 | 0.042436 |
| 72 | V9HWD3 | Epididymis luminal protein 117 | 5 | 2.416 | 0.009535 |
| 73 | Q9H477 | Ribokinase | 2 | 2.412 | 0.028297 |
| 74 | Q8WV93 | AFG1-like ATPase | 2 | 2.410 | 0.000242 |
| 75 | C9JFR7 | Cytochrome c | 58 | 2.409 | 0.010164 |
| 76 | Q9P1F3 | Costars family protein ABRACL | 16 | 2.408 | 0.001172 |
| 77 | A8K2F9 | RNA polymerase II associated protein 1 | 4 | 2.402 | 0.000145 |
| 78 | Q6P996 | Pyridoxal-dependent decarboxylase domain-containing protein 1 | 4 | 2.401 | 0.023423 |
| 79 | Q15052 | Rho guanine nucleotide exchange factor 6 | 1 | 2.394 | 0.001627 |
| 80 | Q14765 | Signal transducer and activator of transcription 4 | 2 | 2.393 | 0.009885 |
| 81 | A8K651 | Complement component 1 Q subcomponent-binding protein, mitochondrial | 37 | 2.390 | 0.016190 |
| 82 | F5H610 | 5'-AMP-activated protein kinase subunit beta-1 | 26 | 2.384 | 0.001468 |
| 83 | Q5T765 | Interferon-induced protein with tetratricopeptide repeats 3 | 3 | 2.370 | 0.007698 |
| 84 | V9HWB5 | Inorganic diphosphatase | 18 | 2.363 | 0.004501 |
| 85 | A0A024R493 | EF-hand domain family, member D1 | 53 | 2.357 | 0.000516 |
| 86 | Q9C002 | Normal mucosa of esophagus-specific gene 1 protein | 11 | 2.355 | 0.000369 |
| 87 | Q8IZJ1 | Netrin receptor UNC5B | 1 | 2.353 | 0.000683 |
| 88 | D6REM4 | Casein kinase I isoform alpha | 4 | 2.352 | 0.014680 |
| 89 | Q15417 | Calponin-3 | 16 | 2.350 | 0.000930 |
| 90 | E9PGA6 | C1QTNF3-AMACR readthrough (NMD candidate) | 7 | 2.345 | 0.025878 |
| 91 | V9GZ56 | U6 snRNA-associated Sm-like protein LSm4 | 10 | 2.344 | 0.001747 |
| 92 | A0A024R3P9 | Acyl-Coenzyme A binding domain containing 3 | 6 | 2.343 | 0.005415 |
| 93 | Q96HC4 | PDZ and LIM domain protein 5 | 4 | 2.337 | 0.002964 |
| 94 | Q96Q11 | CCA tRNA nucleotidyltransferase 1, mitochondrial | 4 | 2.334 | 0.006966 |
| 95 | O00194 | Ras-related protein Rab-27B | 17 | 2.331 | 0.013005 |
| 96 | B7Z569 | Transcription factor Dp-1 | 4 | 2.324 | 0.005602 |
| 97 | Q71RC2 | La-related protein 4 | 2 | 2.318 | 0.017983 |
| 98 | Q15437 | Protein transport protein Sec23B | 10 | 2.312 | 0.038682 |
| 99 | Q2L6I0 | MHC class I region proline-rich protein CAT53 | 4 | 2.309 | 0.014912 |
| 100 | A0A075B6P0 | NADH dehydrogenase (ubiquinone) complex I, assembly factor 6 | 8 | 2.309 | 0.025095 |
| 101 | B4DL98 | Golgin subfamily B member 1 | 5 | 2.307 | 0.007295 |
| 102 | B4DW97 | RNA helicase | 3 | 2.305 | 0.020884 |
| 103 | P36405 | ADP-ribosylation factor-like protein 3 | 20 | 2.302 | 0.009091 |
| 104 | H0Y4Y4 | EF-hand domain-containing protein D2 | 24 | 2.301 | 0.016153 |
| 105 | Q96G03 | Phosphoglucomutase-2 | 2 | 2.300 | 0.003883 |
| 106 | Q9NV31 | U3 small nucleolar ribonucleoprotein protein IMP3 | 10 | 2.300 | 0.008434 |
| 107 | Q9Y232 | Chromodomain Y-like protein | 3 | 2.299 | 0.015361 |
| 108 | Q9BTE3 | Mini-chromosome maintenance complex-binding protein | 2 | 2.297 | 0.007369 |
| 109 | Q86YS6 | Ras-related protein Rab-43 | 12 | 2.297 | 0.000412 |
| 110 | Q6PKG0 | La-related protein 1 | 5 | 2.296 | 0.011246 |
| 111 | Q9BZX3 | Neuron specific transcription factor | 5 | 2.290 | 0.002095 |
| 112 | Q92879 | CUGBP Elav-like family member 1 | 2 | 2.288 | 0.001464 |
| 113 | Q9Y3C8 | Ubiquitin-fold modifier-conjugating enzyme 1 | 8 | 2.285 | 0.010816 |
| 114 | C0IMJ3 | Periostin isoform thy6 | 63 | 2.278 | 0.001941 |
| 115 | Q9Y5K6 | CD2-associated protein | 4 | 2.277 | 0.005356 |
| 116 | A0A087X0M4 | Kanadaptin | 1 | 2.276 | 0.013309 |
| 117 | O94888 | UBX domain-containing protein 7 | 5 | 2.276 | 0.003846 |
| 118 | Q9H6X2 | Anthrax toxin receptor 1 | 4 | 2.274 | 0.003220 |
| 119 | H7C2W9 | 60S ribosomal protein L31 | 21 | 2.265 | 0.003572 |
| 120 | D6RBZ0 | Heterogeneous nuclear ribonucleoprotein A/B | 26 | 2.263 | 0.000377 |
| 121 | Q96DV4 | 39S ribosomal protein L38, mitochondrial | 2 | 2.261 | 0.005265 |
| 122 | Q96AG4 | Leucine-rich repeat-containing protein 59 | 27 | 2.261 | 0.003085 |
| 123 | A0A5C2GTG7 | IG c1800_heavy_IGHV3-23_IGHD1-26_IGHJ3 | 15 | 2.261 | 0.001092 |
| 124 | Q9NTK5 | Obg-like ATPase 1 | 6 | 2.260 | 0.019226 |
| 125 | Q5U071 | High-mobility group box 2 | 20 | 2.258 | 0.003825 |
| 126 | B4DEJ6 | FAS-associated factor 1 | 3 | 2.257 | 0.015333 |
| 127 | A0A158RFU3 | Parathymosin | 23 | 2.257 | 0.009435 |
| 128 | B2R6U8 | Cleavage and polyadenylation specificity factor subunit 5 | 37 | 2.255 | 0.011767 |
| 129 | P25815 | Protein S100-P | 42 | 2.255 | 0.008474 |
| 130 | A0A0S2Z5K8 | Spermatid perinuclear RNA-binding protein | 3 | 2.255 | 0.003500 |
| 131 | C9JIZ6 | Prosaposin | 10 | 2.250 | 0.000138 |
| 132 | P04406 | Glyceraldehyde-3-phosphate dehydrogenase | 61 | 2.248 | 0.012267 |
| 133 | O00469 | Procollagen-lysine,2-oxoglutarate 5-dioxygenase 2 | 3 | 2.245 | 0.011155 |
| 134 | Q15785 | Mitochondrial import receptor subunit TOM34 | 11 | 2.244 | 0.010970 |
| 135 | Q9UMZ1 | Prothymosin a14 | 14 | 2.240 | 0.004391 |
| 136 | Q6GQQ9 | OTU domain-containing protein 7B | 1 | 2.239 | 0.022356 |
| 137 | A8K5M4 | Non-specific serine/threonine protein kinase | 9 | 2.234 | 0.000822 |
| 138 | Q8N8R3 | Mitochondrial basic amino acids transporter | 8 | 2.233 | 0.000015 |
| 139 | B1AHJ7 | Protein-tyrosine sulfotransferase | 10 | 2.232 | 0.014809 |
| 140 | B4E240 | UBX domain-containing protein 2 | 8 | 2.228 | 0.009404 |
| 141 | M0QZK8 | Gamma-glutamylcyclotransferase | 10 | 2.228 | 0.023879 |
| 142 | B4DNH6 | Perilipin | 15 | 2.228 | 0.003920 |
| 143 | A0A6Q8PGU6 | Vesicle-fusing ATPase | 2 | 2.223 | 0.010598 |
| 144 | A0A1C3PI40 | MHC class I antigen | 9 | 2.221 | 0.001358 |
| 145 | Q8WUX9 | Charged multivesicular body protein 7 | 9 | 2.220 | 0.007110 |
| 146 | A0A0U1RQL8 | Macrophage-capping protein | 40 | 2.214 | 0.014432 |
| 147 | E9PK25 | Cofilin, non-muscle isoform | 63 | 2.213 | 0.000451 |
| 148 | J3KPT4 | TraB domain-containing protein | 3 | 2.211 | 0.031691 |
| 149 | E5RFF3 | Zinc finger transcription factor Trps1 | 1 | 2.206 | 0.029039 |
| 150 | Q8IUR0 | Trafficking protein particle complex subunit 5 | 5 | 2.206 | 0.003511 |
| 151 | Q01081 | Splicing factor U2AF 35 kDa subunit | 16 | 2.206 | 0.002124 |
| 152 | J3QRU8 | ARF GTPase-activating protein GIT1 | 2 | 2.206 | 0.002174 |
| 153 | O95373 | Importin-7 | 2 | 2.201 | 0.014348 |
| 154 | O43889 | Cyclic AMP-responsive element-binding protein 3 | 3 | 2.196 | 0.000568 |
| 155 | Q5CZ91 | Methionine aminopeptidase | 7 | 2.193 | 0.025725 |
| 156 | O00571 | ATP-dependent RNA helicase DDX3X | 21 | 2.190 | 0.011402 |
| 157 | Q6PD74 | Alpha- and gamma-adaptin-binding protein p34 | 5 | 2.190 | 0.007494 |
| 158 | Q6P151 | SNW domain-containing protein 1 | 8 | 2.188 | 0.001792 |
| 159 | Q75MU2 | Eukaryotic translation initiation factor 4H | 31 | 2.187 | 0.010263 |
| 160 | A0A0A0RXZ9 | U51 | 2 | 2.185 | 0.014766 |
| 161 | A0A024R3X4 | 60 kDa chaperonin | 84 | 2.185 | 0.002877 |
| 162 | P55327 | Tumor protein D52 | 39 | 2.185 | 0.013931 |
| 163 | A0A024R120 | Transcription factor CP2 | 7 | 2.183 | 0.016141 |
| 164 | Q8N684 | Cleavage and polyadenylation specificity factor subunit 7 | 14 | 2.181 | 0.019213 |
| 165 | A0A7P0T9I6 | Interferon-induced GTP-binding protein Mx1 | 25 | 2.180 | 0.025772 |
| 166 | A0A140VJE3 | Methionine aminopeptidase 2 | 13 | 2.180 | 0.006709 |
| 167 | C9JHK9 | ATP-binding cassette sub-family F member 2 | 5 | 2.173 | 0.006660 |
| 168 | B4DQJ4 | Nuclear valosin-containing protein-like | 3 | 2.172 | 0.003525 |
| 169 | P80723 | Brain acid soluble protein 1 | 80 | 2.171 | 0.000129 |
| 170 | Q6NSJ5 | Volume-regulated anion channel subunit LRRC8E | 1 | 2.168 | 0.033668 |
| 171 | Q9H2P9 | Diphthine methyl ester synthase | 5 | 2.167 | 0.014684 |
| 172 | A0A140VK94 | RAN binding protein 1 | 22 | 2.167 | 0.008962 |
| 173 | A0PJ47 | SAFB2 protein | 15 | 2.165 | 0.008151 |
| 174 | Q5HYD8 | Procollagen-proline 4-dioxygenase | 6 | 2.165 | 0.009299 |
| 175 | B3KX16 | Cartilage-associated protein | 6 | 2.164 | 0.004283 |
| 176 | P24347 | Stromelysin-3 | 2 | 2.163 | 0.006573 |
| 177 | A0A140VK09 | Testicular secretory protein Li 9 | 15 | 2.161 | 0.003517 |
| 178 | Q53FP3 | Cysteine desulfurase, mitochondrial | 7 | 2.160 | 0.022461 |
| 179 | A0A024RDT5 | Periostin, osteoblast specific factor | 63 | 2.160 | 0.000938 |
| 180 | D3DUE6 | Glyoxylate reductase 1 homolog | 6 | 2.159 | 0.033403 |
| 181 | Q7Z3K3 | Pogo transposable element with ZNF domain | 2 | 2.158 | 0.010613 |
| 182 | A0A0G2JLB3 | Glucosylceramidase | 11 | 2.157 | 0.011235 |
| 183 | B4DVZ5 | Nucleolar autoantigen No55 | 3 | 2.156 | 0.001379 |
| 184 | H3BT71 | RNA-binding motif protein, X chromosome | 22 | 2.156 | 0.001016 |
| 185 | B0QYK0 | RNA-binding protein EWS | 8 | 2.151 | 0.010503 |
| 186 | A0A0S2Z4C3 | Fumarate hydratase, mitochondrial | 50 | 2.150 | 0.004516 |
| 187 | P63241 | Eukaryotic translation initiation factor 5A-1 | 66 | 2.149 | 0.005182 |
| 188 | P48735 | Isocitrate dehydrogenase [NADP], mitochondrial | 54 | 2.149 | 0.001121 |
| 189 | A0A1W2PRU0 | Alpha-endosulfine | 12 | 2.147 | 0.004603 |
| 190 | Q99757 | Thioredoxin, mitochondrial | 9 | 2.145 | 0.007301 |
| 191 | B7Z1Q0 | [Heparan sulfate]-glucosamine N-sulfotransferase | 2 | 2.143 | 0.039095 |
| 192 | Q6P1L8 | 39S ribosomal protein L14, mitochondrial | 6 | 2.143 | 0.002762 |
| 193 | Q6FHZ0 | Malate dehydrogenase | 62 | 2.142 | 0.015997 |
| 194 | P20929 | Nebulin | 0 | 2.140 | 0.005163 |
| 195 | W0G8G7 | MHC class I antigen | 21 | 2.139 | 0.006442 |
| 196 | A0A7I2V4B0 | Integrin alpha-L | 6 | 2.139 | 0.016053 |
| 197 | P16278 | Beta-galactosidase | 4 | 2.137 | 0.007638 |
| 198 | P13473 | Lysosome-associated membrane glycoprotein 2 | 7 | 2.132 | 0.006486 |
| 199 | Q9UII2 | ATPase inhibitor, mitochondrial | 25 | 2.132 | 0.001097 |
| 200 | Q96G25 | Mediator of RNA polymerase II transcription subunit 8 | 8 | 2.132 | 0.042751 |
| 201 | Q16740 | ATP-dependent Clp protease proteolytic subunit, mitochondrial | 28 | 2.131 | 0.004157 |
| 202 | H3BSV8 | Eukaryotic peptide chain release factor GTP-binding subunit ERF3A | 8 | 2.130 | 0.011502 |
| 203 | Q6FHB5 | Heme oxygenase | 13 | 2.130 | 0.002638 |
| 204 | A4QN18 | Protein transport protein sec16 | 6 | 2.130 | 0.002533 |
| 205 | B2RB57 | Ubiquitin-like modifier-activating enzyme ATG7 | 2 | 2.129 | 0.009273 |
| 206 | A0A1U9X8J2 | SKIV2L | 1 | 2.128 | 0.012562 |
| 207 | P35270 | Sepiapterin reductase | 17 | 2.127 | 0.001147 |
| 208 | A0A087WYN9 | ATP-dependent RNA helicase DHX29 | 2 | 2.126 | 0.005729 |
| 209 | B4DY08 | Heterogeneous nuclear ribonucleoproteins C1/C2 | 56 | 2.126 | 0.001738 |
| 210 | Q9Y5L4 | Mitochondrial import inner membrane translocase subunit Tim13 | 37 | 2.123 | 0.009051 |
| 211 | F8W8Z9 | Mitochondrial import receptor subunit TOM5 homolog | 8 | 2.122 | 0.005858 |
| 212 | Q9ULL5 | Proline-rich protein 12 | 1 | 2.121 | 0.008052 |
| 213 | Q86XN4 | RNA adenosine deaminase | 1 | 2.120 | 0.000285 |
| 214 | Q15027 | Arf-GAP with coiled-coil, ANK repeat and PH domain-containing protein 1 | 1 | 2.120 | 0.037484 |
| 215 | P62937 | Peptidyl-prolyl cis-trans isomerase A | 59 | 2.120 | 0.008270 |
| 216 | P29966 | Myristoylated alanine-rich C-kinase substrate | 48 | 2.117 | 0.000970 |
| 217 | J3KN67 | Tropomyosin alpha-3 chain | 71 | 2.117 | 0.001426 |
| 218 | Q9P032 | NADH dehydrogenase [ubiquinone] 1 alpha subcomplex assembly factor 4 | 8 | 2.116 | 0.011331 |
| 219 | A0A024RAS5 | Rho GDP dissociation inhibitor beta | 63 | 2.116 | 0.012320 |
| 220 | A0A1U9X8J5 | SLC39A7 | 3 | 2.116 | 0.004883 |
| 221 | Q8TB61 | Adenosine 3'-phospho 5'-phosphosulfate transporter 1 | 6 | 2.115 | 0.003314 |
| 222 | A6NLN1 | Polypyrimidine tract-binding protein 1 | 35 | 2.112 | 0.003782 |
| 223 | Q6FI03 | G3BP protein | 12 | 2.112 | 0.035531 |
| 224 | Q8N257 | Histone H2B type 3-B | 53 | 2.111 | 0.006276 |
| 225 | O95486 | Protein transport protein Sec24A | 4 | 2.110 | 0.019074 |
| 226 | Q9NQR4 | Omega-amidase NIT2 | 10 | 2.110 | 0.007232 |
| 227 | Q13595 | Transformer-2 protein homolog alpha | 8 | 2.110 | 0.002086 |
| 228 | D6W5Y5 | Cold-inducible RNA-binding protein | 12 | 2.109 | 0.020376 |
| 229 | Q8TEM1 | Nuclear pore membrane glycoprotein 210 | 2 | 2.108 | 0.003670 |
| 230 | Q12771 | p37 AUF1 | 37 | 2.107 | 0.005114 |
| 231 | Q96AG6 | HEATR7A protein | 6 | 2.106 | 0.031163 |
| 232 | A2RRH1 | Amine oxidase | 3 | 2.106 | 0.008286 |
| 233 | Q969E4 | Transcription elongation factor A protein-like 3 | 19 | 2.105 | 0.005287 |
| 234 | Q6FH17 | ADP-ribosylation factor 6 | 18 | 2.105 | 0.005646 |
| 235 | Q68Y91 | Hydroxymethylbilane synthase | 13 | 2.103 | 0.015516 |
| 236 | Q6P587 | Acylpyruvase FAHD1, mitochondrial | 40 | 2.102 | 0.029218 |
| 237 | A0A0S2Z4N8 | Vasodilator-stimulated phosphoprotein isoform 2 | 4 | 2.102 | 0.006208 |
| 238 | A0A2R8YE10 | Vacuolar protein sorting-associated protein 45 | 2 | 2.100 | 0.024096 |
| 239 | H3BU69 | Tyrosine-protein kinase CSK | 19 | 2.100 | 0.005938 |
| 240 | Q15047 | Histone-lysine N-methyltransferase SETDB1 | 1 | 2.098 | 0.004970 |
| 241 | Q5TAW7 | Calcium-binding protein 39 | 4 | 2.096 | 0.045046 |
| 242 | P68366 | Tubulin alpha-4A chain | 53 | 2.090 | 0.021107 |
| 243 | Q9Y6Y7 | HCG2024869 | 4 | 2.090 | 0.005669 |
| 244 | Q6IPT9 | Elongation factor 1-alpha | 50 | 2.088 | 0.011059 |
| 245 | B4DHZ9 | cDNA FLJ59623 | 2 | 2.086 | 0.004838 |
| 246 | P31947 | 14-3-3 protein sigma | 19 | 2.085 | 0.028335 |
| 247 | Q9H981 | Actin-related protein 8 | 3 | 2.085 | 0.034464 |
| 248 | A0A024R8S5 | Protein disulfide-isomerase | 74 | 2.085 | 0.004311 |
| 249 | Q9H444 | Charged multivesicular body protein 4b | 15 | 2.084 | 0.024736 |
| 250 | B5MDV5 | KIAA1244 | 1 | 2.084 | 0.011347 |
| 251 | A0A024R7N1 | GEM interacting protein | 4 | 2.083 | 0.002815 |
| 252 | Q3KQZ1 | Solute carrier family 25 member 35 | 4 | 2.080 | 0.012289 |
| 253 | A0A140VJW2 | Stathmin | 13 | 2.078 | 0.002433 |
| 254 | E9PK91 | Bcl-2-associated transcription factor 1 | 5 | 2.075 | 0.008121 |
| 255 | E9PN10 | Receptor-binding cancer antigen-expressed on SiSo cells | 10 | 2.073 | 0.016711 |
| 256 | Q96CS3 | FAS-associated factor 2 | 11 | 2.073 | 0.004471 |
| 257 | A0A096LNZ9 | Ubiquitin-like protein ISG15 | 38 | 2.072 | 0.027703 |
| 258 | P60866 | 40S ribosomal protein S20 | 42 | 2.072 | 0.007476 |
| 259 | A0A1B0GVD5 | Cathepsin D | 50 | 2.071 | 0.003086 |
| 260 | P42224 | Signal transducer and activator of transcription 1-alpha/beta | 16 | 2.071 | 0.017932 |
| 261 | Q59GA1 | Splicing factor, arginine/serine-rich 10 variant | 28 | 2.070 | 0.003920 |
| 262 | P22626 | Heterogeneous nuclear ribonucleoproteins A2/B1 | 59 | 2.069 | 0.005319 |
| 263 | O60547 | GDP-mannose 4,6 dehydratase | 5 | 2.069 | 0.046345 |
| 264 | Q9HCY8 | Protein S100-A14 | 60 | 2.068 | 0.004742 |
| 265 | A0A024RDQ7 | Mitochondrial translational initiation factor 3 | 3 | 2.067 | 0.002802 |
| 266 | B4DKN1 | Non-specific serine/threonine protein kinase | 2 | 2.067 | 0.007375 |
| 267 | O43143 | Pre-mRNA-splicing factor ATP-dependent RNA helicase DHX15 | 5 | 2.066 | 0.007078 |
| 268 | Q9NRG9 | Aladin | 12 | 2.065 | 0.009015 |
| 269 | J3KQ69 | DNA helicase | 3 | 2.065 | 0.029165 |
| 270 | Q9UKV3 | Apoptotic chromatin condensation inducer in the nucleus | 11 | 2.064 | 0.003569 |
| 271 | I3L0A0 | HCG2044781 | 10 | 2.064 | 0.010410 |
| 272 | P45973 | Chromobox protein homolog 5 | 26 | 2.062 | 0.004055 |
| 273 | O60502 | Protein O-GlcNAcase | 1 | 2.060 | 0.004038 |
| 274 | Q99460 | 26S proteasome non-ATPase regulatory subunit 1 | 3 | 2.060 | 0.043356 |
| 275 | Q9HBH0 | Rho-related GTP-binding protein RhoF | 5 | 2.059 | 0.006681 |
| 276 | Q12929 | Epidermal growth factor receptor kinase substrate 8 | 1 | 2.059 | 0.001013 |
| 277 | B4E226 | cDNA FLJ57287 | 5 | 2.058 | 0.019677 |
| 278 | O75323 | Protein NipSnap homolog 2 | 14 | 2.057 | 0.008144 |
| 279 | B4DLN1 | Mitochondrial dicarboxylate carrier | 20 | 2.054 | 0.007769 |
| 280 | Q641R2 | Oxysterol-binding protein | 4 | 2.054 | 0.009205 |
| 281 | B4DZG7 | ADP-ribosylation factor-like protein 1 | 19 | 2.053 | 0.009238 |
| 282 | Q12792 | Twinfilin-1 | 4 | 2.052 | 0.006282 |
| 283 | A0A0G2JMX7 | Microtubule-associated protein | 3 | 2.051 | 0.028524 |
| 284 | H3BPG7 | Anamorsin | 5 | 2.051 | 0.000882 |
| 285 | A0A1V0E3X9 | TAP1 | 7 | 2.051 | 0.014951 |
| 286 | Q9H1B5 | Xylosyltransferase 2 | 1 | 2.051 | 0.031490 |
| 287 | Q8WU76 | Sec1 family domain-containing protein 2 | 2 | 2.050 | 0.005851 |
| 288 | Q6NXG1 | Epithelial splicing regulatory protein 1 | 7 | 2.050 | 0.044345 |
| 289 | Q9NVF7 | F-box only protein 28 | 6 | 2.048 | 0.015926 |
| 290 | A0A6I8PRN4 | Glutamine--fructose-6-phosphate transaminase | 4 | 2.046 | 0.013178 |
| 291 | A0A024RD80 | Heat shock protein 90kDa alpha | 62 | 2.046 | 0.018000 |
| 292 | A8KA83 | Epididymis secretory sperm binding protein | 34 | 2.046 | 0.006717 |
| 293 | O43615 | Mitochondrial import inner membrane translocase subunit Tim44 | 2 | 2.044 | 0.023173 |
| 294 | Q32Q12 | Nucleoside diphosphate kinase | 63 | 2.042 | 0.006509 |
| 295 | P30405 | Peptidyl-prolyl cis-trans isomerase F, mitochondrial | 19 | 2.041 | 0.002834 |
| 296 | A0A140VJY7 | Thioredoxin domain-containing protein 17 | 24 | 2.040 | 0.002122 |
| 297 | V9HWK0 | Signal recognition particle subunit SRP72 | 10 | 2.040 | 0.016911 |
| 298 | P50402 | Emerin | 14 | 2.040 | 0.002735 |
| 299 | H3BPC4 | SUMO-conjugating enzyme UBC9 | 31 | 2.040 | 0.003442 |
| 300 | B3KWE1 | Histidine--tRNA ligase | 3 | 2.039 | 0.014070 |
| 301 | F8W6I7 | Helix-destabilizing protein | 49 | 2.039 | 0.003211 |
| 302 | B4DHE8 | RNA-binding protein Musashi homolog 2 | 5 | 2.038 | 0.008576 |
| 303 | Q5VT52 | Regulation of nuclear pre-mRNA domain-containing protein 2 | 1 | 2.036 | 0.019228 |
| 304 | A0A0G2JI43 | HLA class II histocompatibility antigen, DM beta chain | 9 | 2.035 | 0.007680 |
| 305 | Q8WVV9 | Heterogeneous nuclear ribonucleoprotein L-like | 4 | 2.034 | 0.002713 |
| 306 | B4DL28 | Grainyhead-like protein 2 | 5 | 2.033 | 0.038915 |
| 307 | Q9Y263 | Phospholipase A-2-activating protein | 2 | 2.033 | 0.012630 |
| 308 | A0A024R611 | Coronin | 21 | 2.032 | 0.003275 |
| 309 | Q5TZP7 | DNA-(apurinic or apyrimidinic site) endonuclease | 38 | 2.032 | 0.015143 |
| 310 | J3QQV2 | Monocarboxylate transporter 4 | 2 | 2.032 | 0.018316 |
| 311 | H0Y449 | Y-box-binding protein 1 | 29 | 2.030 | 0.002601 |
| 312 | Q5T5C7 | Seryl-tRNA synthetase | 4 | 2.029 | 0.013168 |
| 313 | B5MDF5 | GTP-binding nuclear protein Ran | 32 | 2.028 | 0.014575 |
| 314 | D6W5K2 | Thymosin, beta 10 | 12 | 2.027 | 0.000377 |
| 315 | B4DT57 | Serine/threonine-protein kinase 24 | 5 | 2.027 | 0.009620 |
| 316 | P07357 | Complement component C8 alpha chain | 3 | 2.026 | 0.009444 |
| 317 | O14561 | Acyl carrier protein, mitochondrial | 22 | 2.024 | 0.035393 |
| 318 | Q15022 | Polycomb protein SUZ12 | 3 | 2.024 | 0.019890 |
| 319 | P61326 | Protein mago nashi homolog | 29 | 2.023 | 0.004998 |
| 320 | Q5T7C4 | High mobility group protein B1 | 20 | 2.022 | 0.003401 |
| 321 | Q9NXE4 | Sphingomyelin phosphodiesterase 4 | 3 | 2.021 | 0.013455 |
| 322 | Q5JUW8 | Discs, large homolog 3 | 2 | 2.020 | 0.039887 |
| 323 | H3BTA3 | E3 ubiquitin-protein ligase CHIP | 6 | 2.020 | 0.007589 |
| 324 | B1AJY5 | 26S proteasome non-ATPase regulatory subunit 10 | 15 | 2.020 | 0.002266 |
| 325 | Q5SY76 | Kinetochore-associated protein NSL1 homolog | 21 | 2.018 | 0.007701 |
| 326 | H0YBU9 | RNA-binding protein with multiple-splicing | 8 | 2.016 | 0.012436 |
| 327 | Q07820 | Induced myeloid leukemia cell differentiation protein Mcl-1 | 2 | 2.016 | 0.006583 |
| 328 | A0A7I2V584 | Signal recognition particle 54 kDa protein | 3 | 2.015 | 0.014160 |
| 329 | P04792 | Heat shock protein beta-1 | 72 | 2.015 | 0.037101 |
| 330 | A0A1W6C2K7 | MHC class I antigen | 24 | 2.015 | 0.013688 |
| 331 | W6A4U0 | Tetraspanin | 8 | 2.014 | 0.007552 |
| 332 | Q99523 | Sortilin | 3 | 2.013 | 0.004033 |
| 333 | A0A024R0Q0 | Protein SMG9 | 3 | 2.012 | 0.011825 |
| 334 | B3KUB4 | Carbonic anhydrase | 13 | 2.012 | 0.018678 |
| 335 | P52597 | Heterogeneous nuclear ribonucleoprotein F | 28 | 2.011 | 0.004668 |
| 336 | Q99985 | Semaphorin-3C | 1 | 2.011 | 0.034810 |
| 337 | B7Z4V2 | 75 kDa glucose-regulated protein | 53 | 2.010 | 0.014753 |
| 338 | A0A2U3TZV8 | Phosphoinositide phospholipase C | 0 | 2.008 | 0.012303 |
| 339 | A0A024R884 | Tenascin C | 42 | 2.008 | 0.041163 |
| 340 | M0R210 | 40S ribosomal protein S16 | 34 | 2.008 | 0.011880 |
| 341 | A8K9X0 | Protein YIPF | 3 | 2.008 | 0.010108 |
| 342 | Q9NSK0 | Kinesin light chain 4 | 3 | 2.008 | 0.019367 |
| 343 | P07996 | Thrombospondin-1 | 29 | 2.006 | 0.005425 |
| 344 | S4TZI3 | MHC class I antigen | 28 | 2.005 | 0.004620 |
| 345 | Q8NHP8 | Putative phospholipase B-like 2 | 3 | 2.004 | 0.005122 |
| 346 | A0A384NKJ3 | Epididymis secretory sperm binding protein | 25 | 2.002 | 0.022399 |
| 347 | A0A2X0TVZ9 | Phosphoinositide 5-phosphatase | 1 | 2.002 | 0.002271 |
| 348 | A0A590UJS9 | Disks large homolog 1 | 2 | 2.002 | 0.020577 |
| 349 | G3V159 | Hermansky-Pudlak syndrome 5 protein | 3 | 2.002 | 0.001886 |
| 350 | A0A7P0TAQ9 | DnaJ homolog subfamily C member 10 | 4 | 2.001 | 0.005001 |
| 351 | Q53G47 | LUC7-like isoform b variant | 7 | 2.001 | 0.008698 |
| 352 | A0A7I2V5W9 | Polyadenylate-binding protein | 10 | 2.000 | 0.014486 |
| 353 | A0A024R5H0 | Barrier to autointegration factor 1 | 64 | 2.000 | 0.000423 |
| 354 | Q59EC0 | Adenosine deaminase, RNA-specific isoform ADAR-a variant | 7 | 2.000 | 0.012711 |
| 355 | B4DUD7 | transcript variant 4 | 2 | 2.000 | 0.015582 |
| 356 | O94925 | Glutaminase kidney isoform, mitochondrial | 7 | 2.000 | 0.004068 |
| 357 | Q9H792 | Inactive tyrosine-protein kinase PEAK1 | 2 | 2.000 | 0.005385 |
| 358 | P30154 | Serine/threonine-protein phosphatase 2A 65 kDa regulatory subunit A beta isoform | 14 | 2.000 | 0.043870 |
| 359 | Q12860 | Contactin-1 | 2 | 0.651 | 0.039058 |
| 360 | H0UI49 | Laminin, alpha 4 | 21 | 0.633 | 0.007482 |
| 361 | Q9NPG2 | Neuroglobin | 7 | 0.631 | 0.013906 |
| 362 | K7EKY5 | Carbonate dehydratase IV | 8 | 0.591 | 0.017188 |
| 363 | Q9BVQ7 | Spermatogenesis-associated protein 5-like protein 1 | 3 | 0.578 | 0.002188 |
| 364 | Q6UWR7 | Glycerophosphocholine cholinephosphodiesterase ENPP6 | 3 | 0.541 | 0.046745 |
| 365 | B4E0A4 | transcript variant 3 | 83 | 0.529 | 0.001555 |
| 366 | B3KWC2 | Contactin-2 | 1 | 0.523 | 0.010587 |
| 367 | A0A6I8PS19 | Guanine nucleotide binding protein-like 3 | 1 | 0.467 | 0.004570 |
| 368 | B0I1P5 | Kidney ankyrin repeat-containing protein 3 | 2 | 0.429 | 0.010223 |
| 369 | B2R8H4 | Glucose transporter type 4 | 3 | 0.284 | 0.011225 |
