# Supplementary figures and images for: ESF1 and MIPEP proteins promote estrogen receptor-positive breast cancer proliferation and are associated with patient prognosis
Source: Clin Proteomics. 2024 Jul 15;21:50. doi: 10.1186/s12014-024-09502-8 (PMC11247778; doi:10.1186/s12014-024-09502-8)

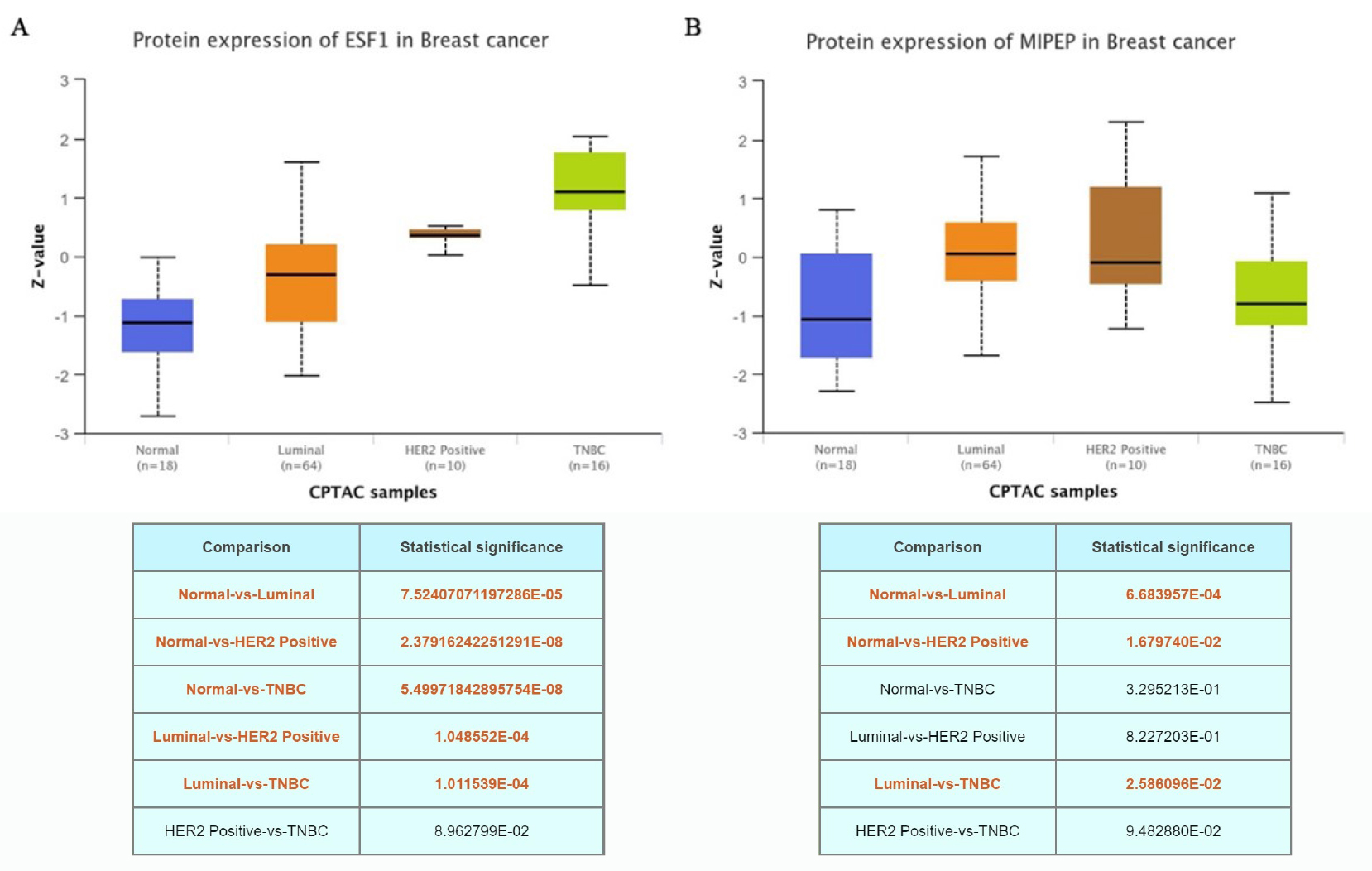

Supplement: Supplementary file 2 — Supplementary Material 2: Fig. 1. Validation of ESF1 and MIPEP proteins expression in an independent cohort. (A) The expression of ESF1 in normal breast tissue (n = 18) and three major molecular subtypes, including luminal ones (n = 64). (B) The expression of MIPEP in normal breast tissue (n = 18) and major subtypes, including luminal ones (n = 64). Z-values represent standard deviations from the median across samples for the given cancer. Log2 Spectral count ratio values from CPTAC were first normalized within each sample profile, then normalized across samples. [file 12014_2024_9502_MOESM2_ESM.jpg]
